# A viability-linked metagenomic analysis of cleanroom environments: eukarya, prokaryotes, and viruses

**DOI:** 10.1186/s40168-015-0129-y

**Published:** 2015-12-08

**Authors:** Thomas Weinmaier, Alexander J. Probst, Myron T. La Duc, Doina Ciobanu, Jan-Fang Cheng, Natalia Ivanova, Thomas Rattei, Parag Vaishampayan

**Affiliations:** Division of Computational Systems Biology, Department of Microbiology and Ecosystem Science, University of Vienna, Vienna, Austria; Department of Earth and Planetary Science, University of California, Berkeley, Berkeley, CA USA; Biotechnology and Planetary Protection Group, Jet Propulsion Laboratory, California Institute of Technology, Pasadena, USA; Precis Scientific, Scottsdale, AZ USA; DOE Joint Genome Institute, Walnut Creek, CA USA

**Keywords:** Indoor microbiome, PMA, Viability, Comparative metagenomics, Spacecraft, Cleanroom, Viruses, Bacteria, Fungi

## Abstract

**Background:**

Recent studies posit a reciprocal dependency between the microbiomes associated with humans and indoor environments. However, none of these metagenome surveys has considered the viability of constituent microorganisms when inferring impact on human health.

**Results:**

Reported here are the results of a viability-linked metagenomics assay, which (1) unveil a remarkably complex community profile for bacteria, fungi, and viruses and (2) bolster the detection of underrepresented taxa by eliminating biases resulting from extraneous DNA. This approach enabled, for the first time ever, the elucidation of viral genomes from a cleanroom environment. Upon comparing the viable biomes and distribution of phylotypes within a cleanroom and adjoining (uncontrolled) gowning enclosure, the rigorous cleaning and stringent control countermeasures of the former were observed to select for a greater presence of anaerobes and spore-forming microflora. Sequence abundance and correlation analyses suggest that the viable indoor microbiome is influenced by both the human microbiome and the surrounding ecosystem(s).

**Conclusions:**

The findings of this investigation constitute the literature’s first ever account of the indoor metagenome derived from DNA originating solely from the potential viable microbial population. Results presented in this study should prove valuable to the conceptualization and experimental design of future studies on indoor microbiomes aimed at inferring impact on human health.

**Electronic supplementary material:**

The online version of this article (doi:10.1186/s40168-015-0129-y) contains supplementary material, which is available to authorized users.

## Background

Over the past decade, numerous studies have reported correlations (of varying strengths and significance) between the microbial communities inhabiting indoor environments and the human microbiome. Most recently, Brooks et al. reported that microbes regularly found in hospitals were capable of colonizing infant guts and could profoundly affect human health [1]. In addition, 16S rRNA gene analysis has been used to show that indoor environments accumulate potential human pathogens in much greater numbers than their surrounding outdoor environments [2]. However, the composition of a given indoor microbiome has also been reported as being strongly influenced by both the architecture and control parameters (e.g., humidity, temperature, airflow, ventilation) of that particular facility [3]. Capitalizing on antimicrobial attributes inherent in architectural design and control logistics is relevant and important to numerous industries, from hospitals to pharmaceutical, microprocessor, and spacecraft manufacturing.

Spacecraft hardware is assembled in controlled cleanroom environments. External to the actual cleanroom, there is an uncontrolled gowning area, i.e., a room in which personnel change into cleanroom garments and make preparations to enter the cleanroom. Due to the elevated extent of human activity, this enclosure is thought to be strongly influenced by the human microbiome. The cleanroom itself has previously been posited as representing an extreme environment [17], characterized by rigorous cleaning and bioburden control regimens, controlled humidity (45 ± 5 %) and temperature (25° C), and a paucity of available nutrients. As a proactive measure to monitor cleanliness and ensure mission integrity, researchers have been diligently cataloging the diverse microbial populations detected about spacecraft and their assembly facilities for decades [4]. Therefore, the indoor microbiome pertaining to spacecraft assembly cleanrooms represents one of the best-studied indoor microbiomes in the literature. The microbial signatures held in this collection were recovered by both cultivation and 16S rRNA gene sequencing [5–11]. As is the case for many other environmental settings, cultivation-based analyses lack the resolution required to capture the entire breadth of microbial diversity housed in indoor environments. It has been estimated that a mere fraction of all microorganisms on Earth are capable of being cultivated in the laboratory [12]. This is due, in large part, to an insufficient understanding of microbial metabolism, interactions (e.g., quorum sensing, symbiosis), and dormancy (e.g., viable but not cultivable status). Ribosomal RNA gene sequence analysis allows for a much higher resolution of microbial diversity profiles than cultivation, despite being limited by primer bias and the generation of phylogenetic information only (no direct metabolic inference). Consequently, environmental genomics based on nucleic acid targets has become an attractive technique for maximizing the coverage of microbial community profiles from indoor environments [13]. However, these DNA-based techniques are incapable of distinguishing viable from dead microbial cells in the samples [14].

Controlled indoor microbiomes are influenced by several factors, including but not limited to routine facility maintenance and cleaning regimens, periodic acute bioburden reduction efforts (e.g., UV lights, vapor-phase H_2_O_2_), controlled humidity and temperature, and a paucity of available nutrients. Consequently, not all microbes can withstand the conditions they encounter in such environments. Recently, the findings of a 16S rRNA gene amplicon study conducted on cleanroom samples suggested that less than 10 % of the observed microbial signatures originated from living microorganisms [11]. This work exploited the viability marker propidium monoazide (PMA), which is able to enter only microbial cells that have a compromised cell membrane [14]. Once inside the compromised cell, PMA binds covalently to DNA molecules, thereby precluding downstream PCR amplification and detection. Previous studies convincingly demonstrated that surveys on microbiomes targeting nucleic acid signatures (e.g., 16S rRNA gene amplicon analysis or metagenomics) sans live/dead chemical markers fail to provide any information on the physiology or viability of the microorganisms from which the detected nucleic acids originated [10, 15, 16]. Consequently, metagenomic analyses based on total environmental DNA extracts do not render a meaningful understanding of the metabolic and/or functional characteristics of living microorganisms in indoor environments.

To overcome this hurdle in indoor microbiome research, we augmented, for the first time ever, metagenomic sequencing with the PMA-based viability assay. This enabled a comprehensive examination of the versatile genetic potential of living biological communities in indoor environments. The results and inferences generated in this study underscore the importance of live/dead chemical markers in studying controlled ecosystems. The experimental design and impactful insights presented here empower the conceptualization and execution of ongoing and future investigations of the indoor microbiome and its impact on human health.

## Results and discussion

### The viable indoor metagenome encompasses eukaryotes, bacteria, and viruses

**Fig. 1:**
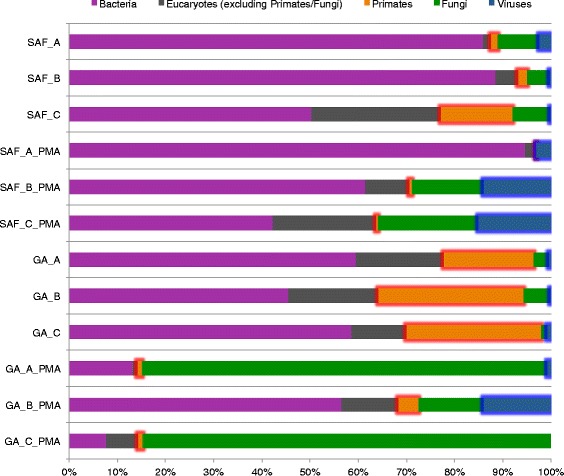
Proportional abundances per sample. Proportional abundances of community subpopulations (bacteria, eukaryotes excluding primates/fungi, primates, fungi, viruses) in different samples. Subpopulations showing a significant change between sample groups are highlighted with a *colored frame*

We analyzed and compared the total biome, and viable contingent thereof, associated with a spacecraft assembly facility. The facility that was examined housed an uncontrolled gowning area and a Class 100K (ISO-8) cleanroom environment. In total, the metagenomes of 12 samples were comparatively analyzed. Three samples were collected from gowning area and three samples were collected from the cleanroom. Each of these samples was split into two equivalent fractions, one of which underwent direct DNA extraction while the other was treated with PMA prior to nucleic acid isolation. Once inside the cell, PMA intercalates and covalently binds to DNA molecules, thereby inhibiting subsequent amplification and/or manipulation of DNA from that particular cell [14]. The taxonomic assignments corresponding to the high-quality reads (Additional file 1: Table S1) populating the metagenomes elucidated in this investigation spanned bacteria, eukaryotes, and viruses (Fig. 1). While the major fraction of most of the resulting metagenomes was attributed to bacteria, two PMA-treated samples collected from the gowning area were mostly populated by fungal sequences (Fig. 1). Non-PMA-treated samples from the gowning area showed a very small proportion of fungal sequences, although between 40 and 50 % of those detected were of primate or other eukaryotic origin. These are likely the remains of dead cells from the human skin and the environment surrounding the facility (e.g., plant cells). A comparison revealed that the primate sequences were significantly more abundant in the non-viable biome compared to the viable biome of both cleanroom and gowning area samples. The presence of viral sequences, on the other hand, was substantially greater in the viable biome. This indicates that the removal of (eukaryotic) DNA from dead cells by PMA treatment enabled the detection of low abundance viruses, which were not detected otherwise.

No archaeal signatures were observed in the original metagenomic dataset. While archaea are known to colonize human skin and are thus readily introduced to indoor environments via shedding [18], the impact of their presence in spacecraft-associated cleanroom environments may have been overestimated in the past [6, 10, 19]. To date, studies have failed to show any evidence in support of archaea actively contributing to cleanroom environments, or posing any threat to cleanroom endeavors [18]. At this time, therefore, archaea cannot be viewed as constituting a significant portion of the cleanroom microbiome.

Taxonomic assignments of metagenomic reads were compared to those presented in Mahnert et al. [26], a study based on 16S rRNA amplicon sequencing of the very same samples (Additional file 2: Table S2). In both studies, Acinetobacter spp. were observed in very high abundance in the spacecraft assembly facility (SAF) and gowning area (GA) samples. Also congruent between the two investigations was the elevated abundance of staphylococcus signatures in GA samples. The high abundance of Bacilli in SAF samples observed in the current study was not reported by Mahnert and co-workers. The differences in signature composition recorded between the two studies likely stem from subtle differences in sample preparation, possible primer bias in the PCR reactions, and the sampling of viral as well as eukaryotic DNA in the metagenomic analyses. While 16S rRNA gene amplicon sequencing can detect low abundant species like Archaea, metagenomic approaches are able to resolve a much more comprehensive understanding of the cleanroom biome, particularly abundant community members.

### Genome reconstruction provides first ever evidence for the presence of viruses in the cleanroom environment

The taxonomic analysis of the metagenomes generated in this study identified a number of different viruses present in the samples. Two phages were detected, a Phi29-like virus and an unclassified Siphoviridae. In addition, several viruses associated with humans or other eukaryotes were detected, namely human herpesvirus 4, Cyclovirus TN12, Dragonfly cyclovirus 2, Hypericum japonicum-associated DNA virus, various Fecal-associated gemycircularviruses, and the Meles meles fecal virus.

**Fig. 2:**
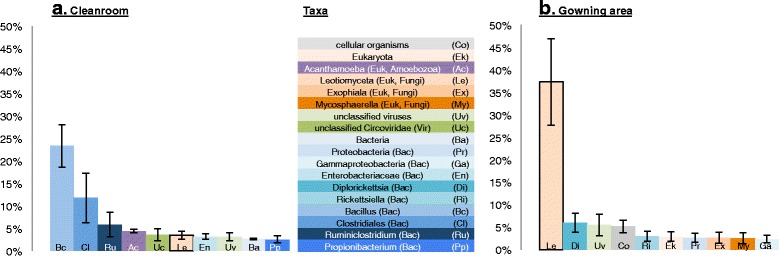
Ranked relative abundances. Rank-abundance curves of relative abundance data in SAF_PMA (**a**) and GA_PMA (**b**) samples. Absolute abundance of each taxon was normalized based on the total abundance of all samples considered. The top ten taxa are listed. *Error bars* indicate standard deviation. Rank-abundance curves for additional sample groups are shown in Additional file 8: Figure S4

The observation of viral signatures inspired further investigation. All datasets were compared to known viral genomes and all of the sequences matching any of those viruses were re-assembled. This was performed separately on each of the two facility areas examined (cleanroom and gowning area). For each of these environments, a subset of the resulting assembly showed high similarity to one known viral genome. Phylogenetic trees were computed based on capsid protein sequences to confirm taxonomic assignments (Additional file 3: Figure S1, 1B and 2B). Sequences reconstructed from the cleanroom samples dataset matched human cyclovirus 7078A, providing average coverage at a level of 3880 across this organism’s entire 1.7 Kb genome (Additional file 3: Figure S1, 1A). The assembly reconstructed from the gowning area samples dataset was highly similar to the genome of *Propionibacterium* phage P14.4 (unclassified Siphoviridae), covering *ca.* 60 % of this virion’s 29 Kb genome at an average coverage of 57 (Additional file 3: Figure S1, 2A). As propionibacterium phages have recently been reported as being abundant on human skin [20], the recovery of such genomes from the gowning area signify the influence of the human skin microbiome on this ecosystem. The presence of genomes from members of the family Circoviridae (Cyclovirus TN12, Dragonfly cyclovirus 2) in the viable metagenome of the cleanroom suggests that human-associated viruses are in fact present in these facilities. Circoviridae was even found to be among the most abundant taxa in the samples (Fig. 2). This finding is of consequence to those managing and maintaining pharmaceutical cleanrooms and hospital operating theaters. The primary objective of these facilities is to prevent the transfer of potential pathogenic organisms, be it via aerosols, fomites, surgical instruments, or medications. As human cycloviruses are frequently involved in disease [21], their observed presence in the cleanroom environment presents an unappreciated potential risk to human health in these types of facilities.

The increased incidence of viral detection in PMA-treated samples is an intriguing finding, one which suggests that PMA preferentially selects for virions having an intact capsid. Another possibility is that certain phages incorporated themselves into the genomes of viable microorganisms as prophages. If this were indeed the case, however, one would expect to observe an elevated infection rate in the microorganisms that were viable. Unless demonstrated otherwise, the authors opine that such a phenomenon would stand in stark contrast to the actual function of viruses (infection and killing of the host). Ergo, we conclude that PMA treatment likely favors the detection of virions with intact capsids.

### Indoor biomes are influenced by both the surrounding ecosystem and the human microbiome

Evaluating the bacterial diversity associated with cleanrooms via sequencing of 16S rRNA genes has led to two strong yet opposing opinions. Initial analyses of geographically distinct cleanrooms suggested that associated microbiomes were largely dependent on the surrounding ecosystem [5, 22, 23]. However, recent studies have claimed more and more congruency between the cleanroom microbiome and the human microbiome, though concrete evidence beyond 16S rRNA gene profile similarity remains elusive [7, 24, 25]. Considering that variation exists in the human skin microbiome due to differences in the biogeographical characteristics of people [20], the observed geographic dissimilarity of cleanroom microbiomes could be attributed to variability resulting from different personnel working in the cleanrooms.

**Fig. 3:**
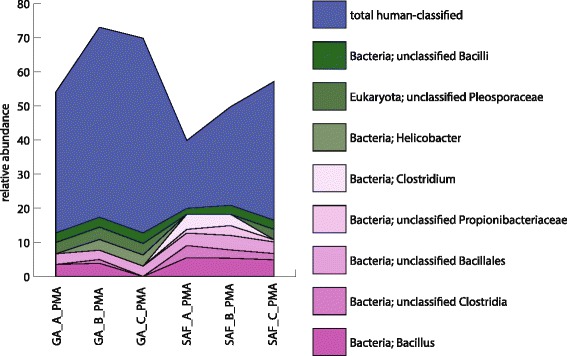
Microbial taxa that significantly correlate with human signals across metagenomes. Relative abundance of human sequences and eight microbial taxa whose abundances were significantly correlated with the abundance of human sequences in gowning area and cleanroom samples, respectively. Unclassified Bacilli, unclassified Pleosporaceae, and Helicobacter spp. showed a positive correlation with the relative abundance of detected human reads, whereas Clostridium, unclassified Propionibacteriaceae, unclassified Bacillales, unclassified Clostridia, and Bacillus spp. showed a negative correlation with such reads

The authors hypothesized that certain viable microbial taxa were dependent on the co-presence of human signatures. To test this, the abundance of human sequences in non-PMA-treated samples was correlated with the abundance of non-human taxa in PMA-treated samples. Results showed a statistically significant correlation between relative human abundance and eight microbial lineages (seven bacterial and one fungal; Spearman correlation, *p* value <0.05), as depicted in Fig. 3. As would be expected, the abundance of human signatures was highest in the gowning area samples and declined in SAF samples. Helicobacter, unclassified Bacilli, and Pleosporaceae exhibited a positive correlation with human signatures, and as such, the authors opine that these organisms are likely introduced to the cleanroom facility via human activity (these organisms were also more abundant in gowning area samples than cleanroom samples). Five bacterial taxa, unclassified Bacillales, Bacillus, unclassified Clostridia, Clostridium, and Propionibacteriaceae showed a negative correlation with human signature abundance. These organisms are therefore likely entering the facility aboard soil and/or dust particles or aerosol droplets that originate in the surrounding external environment. Interesting is the case for *Propionibacterium* spp. cells, which are predominantly anaerobic and thus susceptible to cleanroom conditions. These microbes likely die off shortly after being shed from the skin of their human host, their natural ecosystem, and exposed to oxygen. In concert, the results discussed above clearly demonstrate that (a) the cleanroom microbiome is influenced by both the ecosystem surrounding the facility and the human microbiome, and (b) microorganisms are in fact introduced to cleanroom facilities by humans, despite rigorous and stringent facility maintenance and bioburden control measures.

### Functional and taxonomic complexity of the viable cleanroom microbiome

Understanding the functional potential of the biological communities inhabiting cleanrooms is of importance to a number of industries, including medical, pharmaceutical, superconductor, and space exploration. Those charged with creating, imposing, and enforcing planetary protection policies and requirements have recently come to appreciate the resolving power of innovative molecular strategies to taxonomically and functionally characterize the microbial populations associated with the cleanrooms in which spacecraft are assembled [4]. These endeavors help better estimate the risk of transporting life to foreign celestial bodies, as well as the probability of terrestrial microbiota surviving spaceflight and/or another celestial environment.

The variation observed across taxonomic clades and the influences of different ecosystems on the cleanroom microbiome suggest a fairly complex biological community. This is most likely a consequence of stochastic introduction of microorganisms to the cleanroom facility via the surrounding ecosystem and the shedding of skin from different personnel. Generally speaking, the skin microbiome has been shown to be dependent on the biogeography of the individual [20], which adds yet another level of complexity to the cleanroom ecosystem. A rank-abundance curve based on read abundances (Fig. 2) suggested a fairly simple community, with *Bacillus* and *Clostridiales* highly abundant in PMA-treated SAF samples (Fig. 2a) and the fungus *Leotiomyceta* dominant in PMA-treated GA samples (Fig. 2b). However, this analysis was somewhat limited in that it was predicated on genus, i.e., each genus represented numerous organisms, and thus an array of different genomes. For instance, at least 15 and 34 operational taxonomic units were reported for the highly abundant genera *Bacillus* and *Clostridium*, respectively, by another parallel study (data based on 16S rRNA gene amplicons of the very same samples [26]). These genera are thus representative of at least 15 and 34 different genomes. This observed variability in constituent microbial species, coupled with the detection of various highly abundant eukaryotes (Amoebozoa and fungi) having larger and more complex genomes, leads the authors to conclude that the viable contingent of the cleanroom microbiome is considerably more complex than previously estimated [11]. This complexity hampers sequence assembly for genome reconstruction, as has also been observed for the skin microbiome [20]. Future investigations will necessitate substantially deeper sequencing than has been performed here with very recently developed metagenomic tools that allow resolution at strain level [27].

Genetic evidence for fermentative and respiratory processes was inferred from KEGG annotations. Lactate and alcohol dehydrogenases detected in the metagenome may enable growth of microbes under oxygen-limited conditions via substrate-level phosphorylation. Anaerobic respiration was inferred from genes that encode nitrate and nitrite reductases. Energy generation via respiratory processes may have occurred via NADH dehydrogenases, cytochrome oxidases, and ATP synthases annotated in the metagenome.

Carbon metabolism was inferred from the detection of genes encoding enzymes involved in glycolysis and the TCA cycle. These metabolic processes not only generate ATP but also NADH, which is re-oxidized by either fermentative or respiratory processes (see above). Autotrophic metabolisms were inferred from the detected presence of ATP citrate lyase, a key enzyme for carbon fixation in bacteria operating the reverse TCA cycle. Also found were genes annotated as small subunits of the RuBisCO gene. Although this enzyme’s catalytic subunit is localized on the large chain (i.e., encoded on the marker gene), the presence of the small subunit of the most important enzyme in the Calvin-Benson-Bassham cycle suggests that some organisms may be able to fix carbon dioxide via this pathway. In oligotrophic cleanroom environments, the only readily available source of carbon for microbial proliferation is atmospheric CO_2_, rendering carbon fixation a particularly attractive strategy for the continued persistence and outgrowth of contaminant microorganisms. This metabolic capability has previously been reported in a handful of microbes isolated from cleanrooms [7]. With respect to extraterrestrial environments targeted by future space exploration efforts, organic carbon is most likely limited and autotrophy might very well be the only type of metabolism capable of furnishing hitchhiking microbes with the molecular building blocks required to survive and proliferate.

**Fig. 4:**
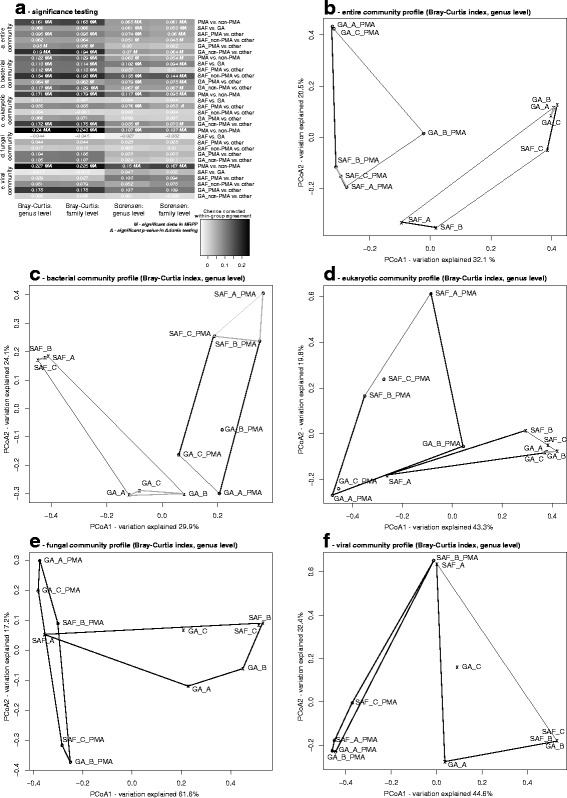
Community composition analyses. **a** Heatmap depicting a summary of the results of PERMANOVA and MRPP tests on the entire community and various subpopulations (bacteria, eukaryotes, fungi, viruses) based on both Bray-Curtis and Sorensen distance. The heatmap is based on the chance corrected within-group agreement of each test, which provides a measure on the intra-group similarity and inter-group dissimilarity (i.e., the higher the value the greater the difference in the community composition between the groups tested). The value is indicated by the cell color, with *white* representing the minimum and *black* representing the maximum value in the heatmap. **b** The observations from significance testing (**a**) were supported by ordination analyses at genus level abundances for entire community profiles. **c** Ordination analysis of bacterial community profiles of all the samples. **d** Ordination analysis of eukaryotic community profiles of all the samples. **e** Ordination analysis of fungal community profiles of all the samples. **f** Ordination analysis of viral community profiles of all the samples

With respect to the reconstruction of metabolic pathways, the elucidation of the total community, even on the sequence level, also remains incomplete. Nevertheless, the partial coverage of some metabolic pathways in the current dataset enabled conclusions regarding stress response, DNA repair, and carbon, nitrogen, and sulfur cycling. The observed abundance of key enzymes in these pathways is depicted in Additional file 4: Figure S2. Key enzyme abundances did not vary significantly between environments (GA vs. SAF). In general, enzyme abundances were higher in samples not treated with PMA. Absolute abundances were particularly high in one sample sans PMA treatment (SAF_A) but dropped below detection limits after the sample had undergone viability treatment (SAF_A_PMA). The placement of these two samples in the community profiles in Fig. 4 suggests that this variation coincides with the eukaryotic fraction of the community. Ultimately, however, only the genes involved in stress response and DNA repair were observed in both the non-PMA-treated and PMA-treated samples. In contrast, genes for carbon, nitrogen, and sulfur cycling were only detected in samples not subjected to the PMA-based viability assay. This might imply that the cells that remain viable in these environments do so by mitigating the stresses imposed with optimally functioning, well-regulated, and DNA repair and stress response pathways. These results underscore the need for more thorough assessments of the functional genetic potential held in the microbial communities dwelling in cleanroom environments.

### Cleanroom maintenance significantly affects microbiome structure

**Fig. 5:**
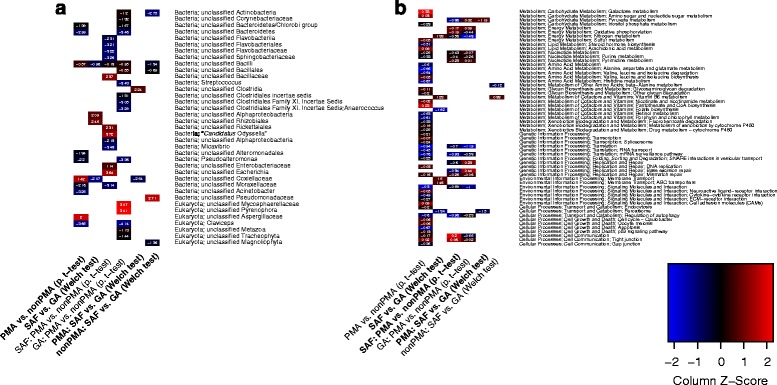
Taxonomic and functional sample differences. Heatmaps displaying differences between the viable and non-viable biome and between gowning area and the cleanroom biome investigated using paired Student’s *t* tests and Welch tests, respectively. These were carried out individually for each classification, namely taxa, KOs (KEGG Orthologs, Additional file 5: Figure S3) and entire KEGG pathways. All tests performed were checked for robustness using permutations of sample sets (Additional file 9: Table S4); robust comparison groups (95 % confidence) were highlighted in this figure and in Additional file 5: Figure S3 (column names of heatmaps). **a** The 37 taxa with significant differences across all six comparison groups comprised Actinobacteria, Bacteroidetes, Firmicutes, Proteobacteria, Fungi, and Viridiplantae. **b** Fifty-eight pathways associated with Metabolism, Genetic Information Processing, Environmental Information Processing, and Cellular Processes showed significant differences between the six comparison groups. Most of the pathways were less abundant in the viable biome

In this study, differences in biome structure were assessed at the single taxon level, at the metabolic pathway level (both see above), and at the community level. When datasets were confined to the eukaryotic and/or viral community, there were no significant differences observed in community structure between the cleanroom and gowning area samples. However, upon analyzing the bacteriome, and even the grand biome in its totality, the cleanroom exhibited a significantly different community composition than the gowning area (Fig. 4). At the taxon level, viable Coxiellaceae and unclassified Clostridia were observed in far greater abundance in the gowning area samples (Fig. 5a). With respect to metabolic pathways, cleanroom samples were significantly depleted of peroxisome and folate biosynthesis pathway signatures yet enriched in nitrogen metabolism, vitamin B6 metabolism, and membrane transport genes (ABC transporters). Certain signatures were markedly underrepresented in the gowning area including an acyltransferase involved in glycerolipid metabolism, a methyltransferase involved in lysine degradation, and several genes associated with genetic information processing (Fig. 5b). These observed differences seem to imply a point at which the cleanroom and gowning area bacteriomes diverged. The authors speculate that differences in the stringency and robustness of cleaning regimens and facility maintenance led to this divergence and continue to impose the varied pressures required to discern these biomes today. These results show that the extent of cleanroom maintenance has a significant influence on the resident viable bacterial community. The same cannot be concluded for the eukaryotic and viral portions of the cleanroom biome.

### The total microbiome and viable contingent thereof have very different taxonomic and functional features

In this investigation, PMA treatment was shown to dramatically alter the structure of recovered biomes, at various levels. At the community level, this viability assay significantly affected the entire, bacterial, eukaryotic, fungal, and viral communities irrespective of the metric applied (binary or abundance; Fig. 4). Permutational MANOVA (PERMANOVA) and multiresponse permutation procedure (MRPP) tests showed high congruency among each other and across the taxonomic groupings tested (genus level and family level). The greatest chance-corrected within-group agreements (Fig. 4a) of all of the tests performed were those for viability assay versus total biome of both fungi and viruses. These two taxonomic groups appeared to be very sensitive to PMA treatment, confirming that PMA-pretreatment did in fact affect the detectability of community members other than bacteria. PMA chemistry has previously been used to discern viable from dead fungi [28, 29] and viruses [30–33]. The authors are aware, however, that PMA-based viability assays are limited in their ability to accurately distinguish viable spores and archaea from their expired counterparts. This limitation might very well explain why no archaea were detected in any of the PMA-treated samples. However, Mahnert et al. detected archaeal signatures at very low abundance in the very same samples via amplicon sequencing [26]. Future experiments might benefit from co-treatment with dithiothreitol, to promote the penetration of PMA into inactivated spores [34]. Whether or not “all” non-viable cells are precluded from downstream molecular detection remains a point of heated debate, and concrete evidence one way or the other continues to elude the PMA community. Although signatures of the human genome decreased significantly after viability treatment (paired student’s *t* test, *p* value 0.025, Fig. 1), some were still found in treated samples. Since it can be assumed that all of the human cells found in these indoor environmental samples are not viable, PMA must sometimes struggle to permeate the (thick) glycocalyx cell walls enveloping these cells. On the other hand, human cells may still have an intact cell wall and thus escape PMA treatment. Nevertheless, the PMA chemistry appeared remarkably effective at manipulating the bias of a molecular reaction (and an entire investigation for that matter) in a favorable manner, i.e., towards the viable community members of interest and away from dead cells and large amounts of human DNA.

On the taxon level (Fig. 5a), the abundance of numerous genera decreased significantly when treated with PMA, while unclassified Aspergillaceae and unclassified Coxiellaceae increased markedly. This effect on Aspergillaceae is of particular interest, as these organisms have been shown to affect human health in indoor environments [34]. The ability to more accurately gauge the abundance of these and other pathogens sans artifacts and bias resulting from the DNA of dead cells should be of interest to health and medical professionals. The authors believe that the enabling capabilities made possible by PMA treatment (resolution of functional, viral, and eukaryotic nucleic acid signatures) largely outweigh the limitations of such treatment on endospores. Therefore, we recommend the augmentation of viability assays as a complement to non-PMA treatment whenever screening for the taxonomic signatures of potentially viable pathogenic organisms. The viable biome resulting from cleanroom samples was far more laden with unclassified Clostridia signatures yet significantly depleted in unclassified Coxiellaceae signatures. This could be a consequence of the physiological flexibility (i.e., anaerobic growth and endospore formation) of Clostridia. The entire (i.e., viable + dead) biome resulting from cleanroom samples also exhibited a reduced abundance of unclassified Coxiellaceae and unclassified Bacilli signatures, whereas the abundance of unclassified Rhizobiales and unclassified Alphaproteobacteria increased significantly.

Changes in functional genetic potential were evaluated at the pathway level (Fig. 5b) while also considering KEGG orthologs (KO; Additional file 5: Figure S3). Functional differences between KO were often observed in pathways whose signature abundances were significantly altered. The detected abundance of most pathway signatures decreased in the viable biome (Fig. 5b, PMA vs. non-PMA). Focusing on cleanroom samples, the viability assay resulted in a slight increase in cell communication signatures and a marked decrease in genes involved in regulation of autophagy, signaling, genetic information processing, and pyruvate and nucleotide metabolism (Fig. 5b, SAF: PMA vs. non-PMA). Of all of the PMA-treated samples analyzed, cleanroom samples were markedly depleted in peroxisome and pyruvate metabolism pathways (Fig. 5b, PMA: SAF vs. GA).

## Conclusions

In conclusion, the results of analyses of taxonomic and functional variability indicate that the gowning area harbors more strictly aerobic and non-spore-forming taxa, while the cleanroom is richer in facultative and obligate anaerobes and spore-forming taxa. These results are in good agreement with findings presented in [26]. Also, the functional profile of the cleanroom biome suggests that this population might be less dependent on oxygen for energy generation and slightly more amenable to other sources, such as nitrogen. Focusing on the viable portion of the microbial community is advantageous for many reasons. Quelling the DNA molecules originating from dead cells imposes a bias in favor of detecting signatures arising from the viable cells of interest. This is of immense importance as researchers attempt to accurately infer microbiome composition and/or function from a given biotope. In natural environments not undergoing drastic changes, the majority of microorganisms exist in an active, viable state [35], while the majority of signatures recovered from indoor microbiomes, cleanrooms in particular, originate from non-viable microorganisms [11]. Understanding the natural status (i.e., viable vs. non-viable) of source organisms is crucial when inferring risk to human health from environmental samples (intensive care units; [36]) via nucleic acid-based analyses. Results convincingly demonstrate that the cleanroom microbiome consists of bacteria, eukaryotes, and even viruses, and as such, is much more complex than was previously posited. Adding to this complexity, at least in part, is an appreciable reciprocal dependency on the human microbiome. The work described here provides a well-established infrastructure for future studies centered on the indoor microbiome and should prove of significant relevance to those interested in epidemiology, pharmaceutical manufacturing and packaging, and operating theater cleanliness or human health in general. Collectively, the experimental design, molecular techniques, and conclusions discussed here constitute the scientific literature’s first ever functional and taxonomic characterization of the viable indoor biome.

## Methods

### Sample collection

Samples were collected from floors of the Jet Propulsion Laboratory’s Spacecraft Assembly Facility (SAF; Pasadena, CA) and adjacent gowning area (GA) via wet surface wiping with biological sampling kits (BiSKit; QuickSilver Analytics, Abingdon, MD), as previously described [37]. In total, ten samples were collected from the SAF and three samples were collected from the adjacent GA (1 m^2^ each), all in triplicate fashion. Negative controls (Sterile PBS prewash of all Sampling kits), handling controls (sampling kits briefly exposed to the ambient sampling environment), and other reagent controls (PBS, DNA extraction reagents) were also prepared. None of the control samples yielded enough DNA to construct metagenome libraries. Hence, these control samples were not considered for sequencing any further analysis. The SAF is a Class 100K certified cleanroom per Fed-Std-209E (equivalent ISO 14644-1 Class 8), within which, spacecraft hardware was actively being assembled at the time that samples were collected.

To minimize microbial contamination of the SAF floor, an all-purpose cleaning and degreasing agent (Kleenol 30, Accurate Industrial Supply, Inc., Cerritos, CA, Cat #: J-CC-00040) is routinely applied by maintenance personnel. Cleanroom surfaces were cleaned twice a day while spacecraft hardware was present and undergoing assembly. In addition, the cleanroom portion of the facility was maintained with stringent protocols geared towards minimizing the influx of particulate matter, including HEPA filtration, the routine replenishment of tacky mats at points of ingress/egress, and daily vacuuming and mopping of floors. Prior to entering the cleanroom, personnel were required to take necessary precautions in the gowning area, including the donning of cleanroom garments, the gloving of hands, and the taping of gloves to garments. Hence, the gowning area was also sampled as a means of evaluating the extent to which microbes gain entry into the cleanroom via this portal.

### Sample processing

Sample volumes were extracted from each BiSKit device in accordance with manufacturer-provided protocols. Biological materials from each 45 ml sample were concentrated with Amicon Ultra-50 Ultracel centrifugal filter tubes (Millipore, Billerica, MA). Each filter unit, having a molecular mass cutoff of 50 kDa, facilitated the concentration of cells, spores, and nucleic acid fragments greater than 100 bp. All concentrated samples (1 ml final) were divided into two separate 500 μL fractions, one to be treated with PMA prior to analysis (viability assessment), and the other to serve as a null environmental sample (viable + non-viable, i.e., total DNA).

### Viability assay

Each 500 μl aliquot of filter-concentrated sample suspension to undergo viability assessment was treated with PMA (2 mM; Biotium, Inc., Hayward, CA) to a final concentration of 50 μM [16, 38], mixed thoroughly, and incubated in the dark for 5 min at room temperature. Tubes were inverted 5–6 times manually during the incubation to promote homogeneous PMA exposure. Both PMA-treated and non-treated samples were subjected to PMA photoactivation at room temperature for 15 min using a LED light source (*λ* = 464–476 nm, 60 W; PhAST Blue, GenIUL, Barcelona, Spain). To facilitate recovery of the broadest spectrum of recovered DNA molecules possible, one-half of the volume of each sample (250 μl) was subjected to bead beating in Lysing Matrix E tubes (60 s at 10 m/s) on a FastPrep®-24 (MP Biomedicals, Solon, OH, USA). Following agitation, respective sample fractions were combined (500 μl) and subjected to automated DNA extraction in a Maxwell® 16 instrument, in accordance with the manufacturer’s accordance with mPromega; Madison, WI). The DNA extracts resulting from the ten cleanroom samples were then pooled, as were those from the three gowning area samples. As samples were collected in triplicate from each sampling location, processing in this manner resulted in three representative samples each from the cleanroom and gowning area.

### Metagenomic sequencing

All manipulations were performed in a bleach-cleaned biohood, which resided in an ultra-clean laboratory environment (i.e., single-use lab coats, bleached gloves, booties, etc.). Each sample was divided into 1 μl aliquots, which were amplified via Multiple Displacement Amplification (MDA) using Repli-g single-cell whole genome amplification kit (Qiagen part #150345) according to the manufacturer’s instructions. Reaction mixture consisted of Phi29 Reaction Buffer (1X final concentration), 50 ng in hexamers with phosphoro- thioate modification of the two 3’-terminal nucleo-tides (IDT) [39], 0.4 mM dNTP, 5 % DMSO (Sigma), 10 mM DTT (Sigma), 100 U Phi29, and 0.5 μM Syto 13 (Invitrogen) in a final volume of 15 μl. A master mixture of MDA reagents was prepared and subsequently dispensed into Safe-Lock 1.5 ml clear microcentrifuge tubes (Eppendorf). Syto 13 was omitted from the master mixture as it is easily degraded by UV radiation. All plastic ware, water, lysis, and stop buffer were UV treated in a Stratalinker 2400 UV Crosslinker (Stratagene) with 254-nm UV for 30 to 90 min on ice [40]. This represents a UV dose range of 5.7 to 17.1 J/cm^2^, calculated by measuring the distance from inside the tubes to the light bulb (4 cm). Following UV irradiation, master mixture was augmented with Syto 13 and dispensed into each well of a 384-well plate. MDA reactions were real-time monitored and stopped when sample amplification reached saturation.

Amplified fractions of each sample were combined, and this pooled DNA product (100 μl) was sheared using a Covaris E210 instrument (Covaris, Woburn, MA) set to 10 % duty cycle, intensity 5, and 200 cycles per burst for 1 min. The concentration and fragment size of each sheared product was determined using Agilent 2100 Bioanalyzer (Agilent Technologies, Santa Clara, CA) in accordance with the manufacturer’s recommended conditions. The sheared DNA was end-repaired, A-tailed, and ligated to Illumina adaptors according to standard Illumina (Illumina, San Diego, CA) PE protocols. The concentration of the resulting Illumina-indexed libraries was again determined using Agilent 2100 Bioanalyzer (Agilent Technologies, Santa Clara, CA). JPL samples GA-A, GA-B, GA-C, GA-A + PMA, GA-B + PMA, and GA-C + PMA were pooled into one library; JPL samples SAF-A, SAF-B and SAF-C, SAF-A + PMA, SAF-B + PMA, SAF-C + PMA were pooled into a second library. In this context, “pooling” refers to the barcoding and multiplexing of numerous sample sets into a single library. The pooled libraries were normalized to a final concentration of 400 mM each, and the primary bands corresponding to the sizes were gel-purified and dissolved in 30 μl TE. One flow-cell was generated from each pooled library, which was subsequently subjected to sequencing in an Illumina MiSeq instrument, in accordance with manufacturer-provided protocols. The raw sequence data are available within IMG/M (http://img.jgi.doe.gov/).

### Sequence data analysis

MiSeq-generated paired-end reads 250 bp in length were merged using PEAR software (default parameters) [41], and both the merged reads and each of the non-merged reads (forward and reverse) were retained. FastQC [42] was used to determine the base quality throughout the reads, and all merged and non-merged reads were processed using prinseq-lite [43] with the parameters: “-min_len 100 -trim_qual_right 20-trim_qual_left 20-trim_left 8.” Adapter sequences and overrepresented homooligonucleotides were identified with the tool FastQC [42] and removed using Cutadapt [44]. The remaining high-quality reads were mapped against the genome of the Illumina positive sequencing control, Bacteriophage PhiX174, and a JGI standard collection of potential contaminant genomes (Additional file 6: Table S3) using the BBMap short read aligner [45]. Any reads matching any of these contaminant genomes were removed from the dataset. Remaining high-quality, non-contaminant reads were assembled using the Velvet [46], Ray Meta [47], and IDBAUD [48] assembly tools. The assemblies resulting from each of the three tools were of low value (largest contig, 2–17 Kb; N50, 0.6–1 Kb, coverage, 1–4), and as such, all subsequent analyses were based on unassembled read data.

All high-quality, non-contaminant reads were compared against NCBI non-redundant database (NR) [49] using RAPSearch2 [50], and results were imported into MEGAN (min score, 80; [51]). Read counts per taxon were exported for family and genus level, as were counts for functional assignments against KEGG on KEGG ortholog (KO) and pathway level. This represented the total abundance dataset. For various sample groups, genus level taxon abundances were summed, ranked, and normalized based on the total abundance of all taxa in the respective group of samples. The top ten taxa in each group of samples were then plotted in a rank-abundance curve.

Bacterial taxa were compared to the results from Mahnert et al. [26], which are based on the same samples. Genus level taxon abundances were summed and normalized based on the total abundance of all taxa in the respective samples of both studies, and top 20 taxa for each sample were extracted.

For univariate statistics, human reads were removed from the dataset. Therefore, all high-quality, non-contaminant reads were mapped against the human assembly “GRCh38” (including the mitochondrial genome) using BBMap [45], and matching reads were removed. Remaining reads were compared against NCBI NR [49] using RAPSearch2 [50], and the results were imported into MEGAN (min score, 80; [51]). For each sample, the “primate” sub-branch was removed, and then read counts per taxon, as well as for functional assignment against KEGG on KO and pathway level, were determined as described above. This represented the non-human abundance dataset.

High-quality non-contaminant reads were also mapped against all viral genomes in NCBI RefSeq [49] using BBMap [45]. Reads matching to viral genomes were extracted, grouped by environment (SAF or GA), and assembled with the metagenome assembler Ray Meta [47]. For each environment (SAF or GA), the reads used for assembly were mapped to the resulting contigs to derive coverage and validate the assembly. Assembled contigs were then compared to the NCBI NR database via BlastX [52] and aligned against the genome sequences of the best BlastX hits using MAUVE [53]. Capsid proteins detected by BlastX in each of the contig subsets were aligned to amino acid sequences of capsid homologs in closely related taxa using Muscle [54] with default parameters, and a maximum-likelihood phylogenetic tree was constructed from the alignment with FastTree 2 using default parameters [55].

### Statistical analysis

Taxonomically and functionally classified sequences were analyzed using the R programming environment [56]. Multivariate statistics were based on rarefaction of the non-human abundance dataset to the lowest amount of reads of all samples. Rarefaction, followed by calculating the Bray-Curtis or Sorensen distance, was performed 10,000 times, and the average distance was calculated. Tests using this averaged distance spanned PERMANOVA (Adonis testing), MRPP, and principal coordinate analysis (PCoA) calculated using the R-vegan package [57]. The according R script can be found in the supplementary (Additional file 7: Zipfile S1).

Calculations for determining significantly increased/depleted taxa were based on log10-transformed sequence abundance data (normalized by number of reads) and included paired *t* tests (when pairing was possible due to PMA treatment) and Welch tests for non-paired data (comparisons across non-paired samples, e.g., cleanroom vs. gowning area). Additionally, a permutation test was carried out to check for false discovery. Abundance differences for significant taxa were visualized as a heatmap of Z-scores.

Correlations between abundance of human and every classified taxon were calculated based on the total abundance dataset using Spearman’s correlation coefficient. Taxa within the “Eumetazoa” lineage were excluded from the correlation analysis, as these were likely to represent nonspecific human sequences. Abundance data was log10-transformed and normalized by number of reads. Human abundance per non-PMA sample was determined by summing up abundances of all taxa that contain “Primates” in their lineage. Human abundances in non-PMA samples were correlated with the abundances in PMA samples for each taxon by Spearman’s correlation coefficient.

### Functional analysis

Functional annotations were derived from MEGAN [51]. Significantly increased/depleted pathways and KEGG orthology (KO) were based on log10-transformed non-human sequence abundance data (normalized by number of reads) and were identified by paired *t* tests and Welch tests as described above for taxa. A permutation test was carried out to check for false discovery, and abundance differences for significant pathways/KOs were visualized as a heatmap of Z-scores. The complete set of KO annotations was searched for key terms related to stress response and DNA repair. Search terms used were “sulfoxide,” “thioredoxin,” “homologous,” “repair,” “sbc,” “recombination,” “exopolysaccharide,” “glycosylase,” “heat,” and “cold.” The relative abundance of enzymes annotated with these key terms, as well as enzymes contained in the KEGG pathways “Carbon fixation in prokaryotes,” “Nitrogen metabolism,” and “Sulfur metabolism” were calculated by dividing absolute abundance values by the total number of functional annotations. Coverage of KEGG pathway maps were manually inspected using MEGAN.

### Availability of data and materials

The data sets supporting the results of this article are available within IMG/M (http://img.jgi.doe.gov/).

## Additional files

Additional file 1: Table S1. An Excel table of Sequence statistics. Read count and base pairs for the different step in the sequence processing. (XLSX 16 kb) Additional file 2: Table S2. An Excel table comparing taxonomic annotations to Mahnert et al. [26]. (XLSX 56 kb) Additional file 3: Figure S1. A PDF figure illustrating the reconstructed viral genomes. Whole genome alignment and phylogenetic tree of capsid gene for the two reconstructed viral genomes. A) Whole genome alignment of assembled contigs against the viral reference genome that showed the highest similarity (1A and 2A). Coverage distribution is indicated above alignment on log scale. B) Maximum-likelihood phylogenetic tree for the capsid gene and its closest homologs. (PDF 917 kb) Additional file 4: Figure S2. A PDF showing a heatmap of relative abundances. Heatmap of relative abundances of key enzymes, with respect to planetary protection, among all functional annotations. Blue: minimum values; Red: maximum values. (PDF 50 kb) Additional file 5: Figure S3. A PDF figure showing significantly different KEGG Orthologs (KOs). 35 KEGG Orthologs that showed significant differences between the six comparison groups. (PDF 39 kb) Additional file 6: Table S3. An Excel table listing the contaminant genomes. List of JGI standard set of genomes that represent likely contaminations and therefore were filtered out in the sequence processing. (XLSX 10 kb) Additional file 7: S1. A PDF file containing the R script MCStats1.2. R script for statistical analysis of ecological community data. (DOC 25 kb) Additional file 8: Figure S4. A PDF figure depicting additional ranked abundance curves. Rank-abundance curves of relative abundance data in different sample groups. Absolute abundance of each taxon was normalized by the total abundance of all samples considered. Top ten taxa are listed. Error-bars indicate standard deviation. Proportional abundances of top 20 bacterial taxa (genus level) compared by sample. (PDF 162 kb) Additional file 9: Table S4. An Excel table containing permutation test results. Number of significant taxa/KOs/pathways for paired *t* tests and Welch tests between comparison groups and results for the same tests in 100 random permutations. (XLSX 44 kb)
