# Supplementary figures and images for: A viability-linked metagenomic analysis of cleanroom environments: eukarya, prokaryotes, and viruses
Source: Microbiome. 2015 Dec 8;3:62. doi: 10.1186/s40168-015-0129-y (PMC4672508; doi:10.1186/s40168-015-0129-y)

1A

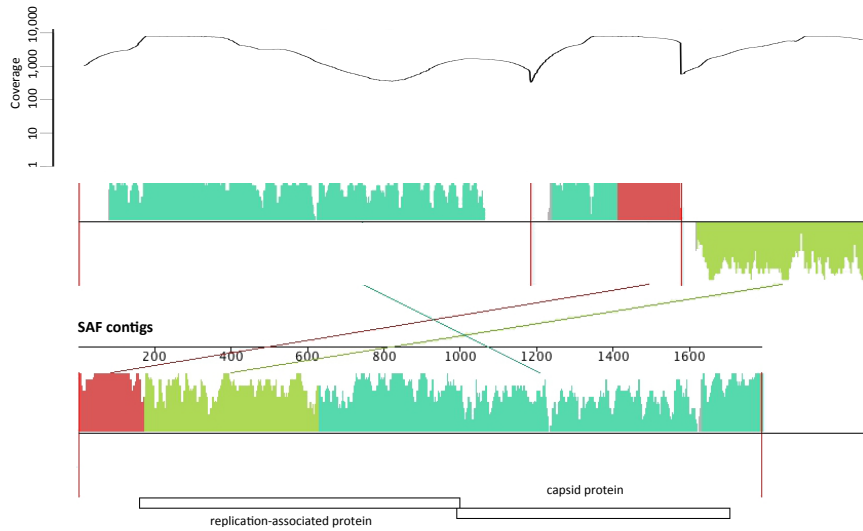

Human cyclovirus 7078A

2A

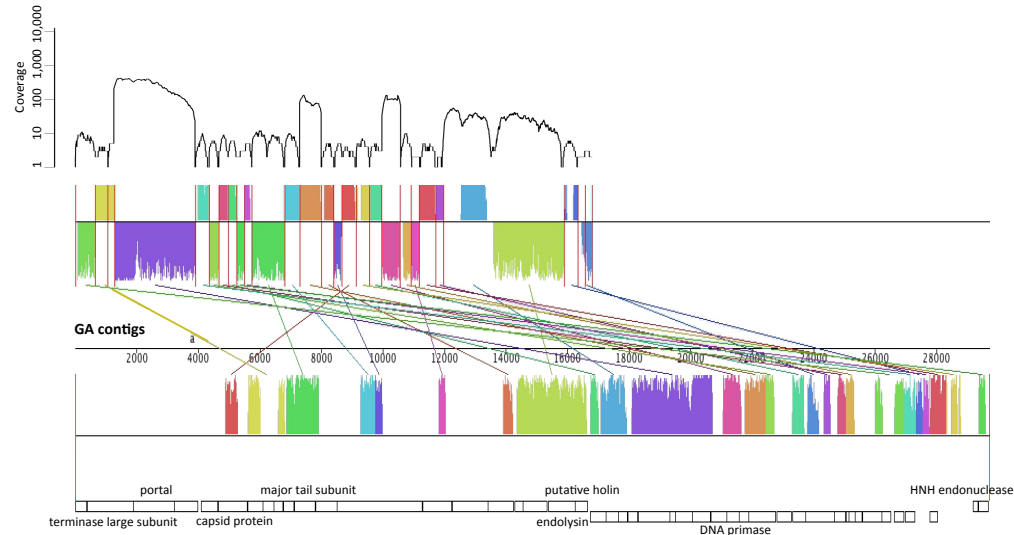

Propionibacterium phage P14.4

1B

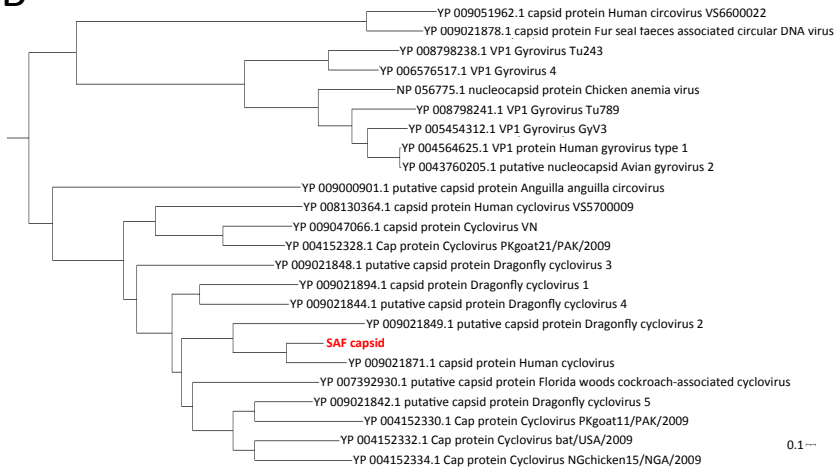

0.1

2B

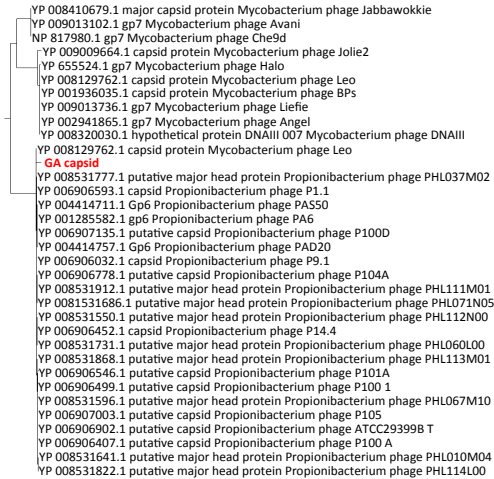

1

Supplement: Additional file 3: Figure S1. — A PDF figure illustrating the reconstructed viral genomes. Whole genome alignment and phylogenetic tree of capsid gene for the two reconstructed viral genomes. A) Whole genome alignment of assembled contigs against the viral reference genome that showed the highest similarity (1A and 2A). Coverage distribution is indicated above alignment on log scale. B) Maximum-likelihood phylogenetic tree for the capsid gene and its closest homologs. (PDF 917 kb) [file 40168_2015_129_MOESM3_ESM.pdf]
