# Supplementary material for: A viability-linked metagenomic analysis of cleanroom environments: eukarya, prokaryotes, and viruses
Source: Microbiome. 2015 Dec 8;3:62. doi: 10.1186/s40168-015-0129-y (PMC4672508; doi:10.1186/s40168-015-0129-y)
Supplement: Additional file 4: Figure S2. — A PDF showing a heatmap of relative abundances. Heatmap of relative abundances of key enzymes, with respect to planetary protection, among all functional annotations. Blue: minimum values; Red: maximum values. (PDF 50 kb) [file 40168_2015_129_MOESM4_ESM.pdf]

		GA_A	GA_B	GA_C	SAF_A	SAF_B	SAF_C	GA_A_PMA	GA_B_PMA	GA_C_PMA	SAF_A_PMA	SAF_B_PMA	SAF_C_PMA
**Heat shock proteins**	heat shock 70kDa protein 1/8	7.2E-05	0.0E+00	0.0E+00	4.1E-05	0.0E+00	0.0E+00	0.0E+00	0.0E+00	0.0E+00	0.0E+00	0.0E+00	0.0E+00
	HSFF; heat shock transcription factor, other eukaryote	0.0E+00	0.0E+00	0.0E+00	0.0E+00	0.0E+00	0.0E+00	0.0E+00	0.0E+00	0.0E+00	0.0E+00	1.3E-05	0.0E+00
	heat shock 70kDa protein 4	0.0E+00	0.0E+00	0.0E+00	8.9E-05	0.0E+00	0.0E+00	0.0E+00	0.0E+00	0.0E+00	0.0E+00	0.0E+00	0.0E+00
	BIP; heat shock 70kDa protein 5	0.0E+00	0.0E+00	0.0E+00	0.0E+00	0.0E+00	0.0E+00	0.0E+00	0.0E+00	0.0E+00	0.0E+00	0.0E+00	2.7E-06
**DNA repair**	DNA-3-methyladenine glycosylase I [EC:3.2.2.20]	0.0E+00	0.0E+00	0.0E+00	4.1E-06	0.0E+00	0.0E+00	0.0E+00	0.0E+00	0.0E+00	0.0E+00	0.0E+00	0.0E+00
	DNA glycosylase [EC:3.2.2.-]	0.0E+00	0.0E+00	0.0E+00	1.2E-05	0.0E+00	3.1E-04	0.0E+00	0.0E+00	0.0E+00	0.0E+00	0.0E+00	0.0E+00
	mutY; A/G-specific adenine glycosylase [EC:3.2.2.-]	0.0E+00	0.0E+00	0.0E+00	0.0E+00	3.6E-03	0.0E+00	0.0E+00	0.0E+00	0.0E+00	0.0E+00	0.0E+00	0.0E+00
	UNG, UDG; uracil-DNA glycosylase [EC:3.2.2.27]	7.2E-05	0.0E+00	0.0E+00	0.0E+00	0.0E+00	0.0E+00	0.0E+00	0.0E+00	0.0E+00	0.0E+00	5.4E-05	0.0E+00
	TDG/mug DNA glycosylase family protein [EC:3.2.2.-]	0.0E+00	0.0E+00	0.0E+00	4.1E-06	0.0E+00	0.0E+00	0.0E+00	0.0E+00	0.0E+00	0.0E+00	0.0E+00	0.0E+00
	OGG1; N-glycosylase/DNA lyase [EC:3.2.2.- 4.2.99.18]	0.0E+00	0.0E+00	0.0E+00	4.1E-06	0.0E+00	1.0E-04	0.0E+00	0.0E+00	1.5E-05	0.0E+00	0.0E+00	0.0E+00
	mutM, fpg; formamidopyrimidine-DNA glycosylase [EC:3.2.2.23 4.2.99.18]	0.0E+00	1.5E-04	0.0E+00	2.8E-05	0.0E+00	0.0E+00	0.0E+00	0.0E+00	0.0E+00	0.0E+00	0.0E+00	0.0E+00
	XPA; DNA-repair protein complementing XP-A cells	0.0E+00	0.0E+00	0.0E+00	0.0E+00	0.0E+00	0.0E+00	0.0E+00	5.2E-05	0.0E+00	0.0E+00	0.0E+00	8.1E-06
	DNA repair protein REV1 [EC:2.7.7.-]	7.2E-05	2.4E-03	0.0E+00	3.7E-05	3.6E-03	1.3E-03	1.3E-04	0.0E+00	7.7E-06	0.0E+00	1.0E-04	1.9E-05
	DNA mismatch repair protein MutS	0.0E+00	0.0E+00	0.0E+00	4.1E-06	0.0E+00	0.0E+00	0.0E+00	0.0E+00	0.0E+00	0.0E+00	0.0E+00	0.0E+00
	DNA mismatch repair protein MLH1	0.0E+00	0.0E+00	0.0E+00	8.1E-06	0.0E+00	0.0E+00	0.0E+00	0.0E+00	0.0E+00	0.0E+00	3.6E-05	0.0E+00
	DNA mismatch repair protein MSH2	0.0E+00	0.0E+00	0.0E+00	5.7E-05	0.0E+00	0.0E+00	0.0E+00	0.0E+00	0.0E+00	0.0E+00	0.0E+00	0.0E+00
	DNA mismatch repair protein MSH3	0.0E+00	0.0E+00	0.0E+00	2.4E-05	0.0E+00	1.0E-04	0.0E+00	0.0E+00	0.0E+00	0.0E+00	0.0E+00	0.0E+00
	DNA mismatch repair protein MSH6	0.0E+00	0.0E+00	0.0E+00	4.1E-06	0.0E+00	0.0E+00	0.0E+00	0.0E+00	0.0E+00	0.0E+00	0.0E+00	0.0E+00
	DNA excision repair protein ERCC-1	0.0E+00	0.0E+00	0.0E+00	0.0E+00	0.0E+00	1.0E-04	0.0E+00	0.0E+00	0.0E+00	0.0E+00	0.0E+00	0.0E+00
	XPB; DNA excision repair protein ERCC-3 [EC:3.6.4.12]	0.0E+00	0.0E+00	1.5E-03	1.2E-05	0.0E+00	0.0E+00	0.0E+00	0.0E+00	0.0E+00	0.0E+00	0.0E+00	0.0E+00
	XPG, RAD2; DNA excision repair protein ERCC-5	0.0E+00	0.0E+00	0.0E+00	3.8E-04	0.0E+00	0.0E+00	0.0E+00	0.0E+00	0.0E+00	0.0E+00	0.0E+00	0.0E+00
	CSB, RAD26; DNA excision repair protein ERCC-6	0.0E+00	1.5E-04	0.0E+00	0.0E+00	0.0E+00	0.0E+00	0.0E+00	0.0E+00	0.0E+00	0.0E+00	0.0E+00	0.0E+00
**Homologous recombination**	DNA repair and recombination protein RAD54 and RAD54-like protein [EC:3.6.4.-]	0.0E+00	0.0E+00	0.0E+00	3.3E-04	0.0E+00	0.0E+00	0.0E+00	0.0E+00	0.0E+00	0.0E+00	2.6E-06	0.0E+00
	DNA repair and recombination protein RAD54B [EC:3.6.4.-]	0.0E+00	0.0E+00	0.0E+00	3.4E-04	0.0E+00	0.0E+00	0.0E+00	0.0E+00	0.0E+00	0.0E+00	0.0E+00	0.0E+00
	DNA repair protein RAD57	7.2E-05	0.0E+00	0.0E+00	0.0E+00	0.0E+00	0.0E+00	0.0E+00	0.0E+00	0.0E+00	0.0E+00	0.0E+00	0.0E+00
	recombination protein RecR	0.0E+00	0.0E+00	0.0E+00	4.1E-06	0.0E+00	0.0E+00	0.0E+00	0.0E+00	0.0E+00	0.0E+00	0.0E+00	0.0E+00
	DNA recombination protein RmuC	0.0E+00	0.0E+00	0.0E+00	0.0E+00	0.0E+00	0.0E+00	0.0E+00	5.2E-05	0.0E+00	0.0E+00	0.0E+00	0.0E+00
**Carbon fixation**	3-hydroxyacyl-CoA dehydrogenase [EC:1.1.1.35]	0.0E+00	0.0E+00	0.0E+00	1.6E-05	0.0E+00	0.0E+00	0.0E+00	0.0E+00	0.0E+00	0.0E+00	0.0E+00	0.0E+00
	Methylenetetrahydrofolate reductase (NADPH) [EC:1.5.1.20]	0.0E+00	0.0E+00	0.0E+00	0.0E+00	0.0E+00	0.0E+00	0.0E+00	0.0E+00	7.7E-06	0.0E+00	0.0E+00	0.0E+00
**Nitrogen metabolism**	Cyanate lyase [EC:4.2.1.104]	0.0E+00	0.0E+00	0.0E+00	0.0E+00	0.0E+00	0.0E+00	0.0E+00	0.0E+00	7.7E-06	0.0E+00	0.0E+00	0.0E+00
	Carbonic anhydrase [EC:4.2.1.1]	0.0E+00	0.0E+00	0.0E+00	1.6E-05	0.0E+00	0.0E+00	0.0E+00	0.0E+00	0.0E+00	0.0E+00	0.0E+00	0.0E+00
	Carbonic anhydrase [EC:4.2.1.1]	0.0E+00	0.0E+00	1.5E-04	4.1E-06	0.0E+00	0.0E+00	0.0E+00	0.0E+00	0.0E+00	0.0E+00	0.0E+00	0.0E+00
	Carbonic anhydrase [EC:4.2.1.1]	0.0E+00	0.0E+00	0.0E+00	0.0E+00	0.0E+00	3.1E-04	0.0E+00	0.0E+00	0.0E+00	0.0E+00	0.0E+00	0.0E+00
	Aminomethyltransferase [EC:2.1.2.10]	7.2E-05	0.0E+00	0.0E+00	1.2E-04	0.0E+00	0.0E+00	0.0E+00	0.0E+00	0.0E+00	0.0E+00	0.0E+00	0.0E+00
	L-asparaginase [EC:3.5.1.1]	0.0E+00	0.0E+00	0.0E+00	4.1E-06	0.0E+00	0.0E+00	0.0E+00	0.0E+00	0.0E+00	0.0E+00	0.0E+00	0.0E+00
	Nitronate monooxygenase [EC:1.13.12.16]	7.2E-05	0.0E+00	0.0E+00	0.0E+00	0.0E+00	0.0E+00	0.0E+00	0.0E+00	0.0E+00	0.0E+00	0.0E+00	0.0E+00
	Nitrate reductase (NADPH) [EC:1.7.1.3]	0.0E+00	0.0E+00	0.0E+00	1.6E-05	0.0E+00	0.0E+00	0.0E+00	0.0E+00	0.0E+00	0.0E+00	0.0E+00	0.0E+00
	Nitrite reductase (NAD(P)H) large subunit [EC:1.7.1.4]	0.0E+00	0.0E+00	0.0E+00	4.1E-06	0.0E+00	0.0E+00	0.0E+00	0.0E+00	0.0E+00	0.0E+00	0.0E+00	0.0E+00
	Glutamate dehydrogenase [EC:1.4.1.2]	0.0E+00	0.0E+00	0.0E+00	2.6E-04	0.0E+00	0.0E+00	0.0E+00	0.0E+00	0.0E+00	0.0E+00	0.0E+00	0.0E+00
	Glutamate dehydrogenase (NAD(P)+) [EC:1.4.1.3]	0.0E+00	0.0E+00	0.0E+00	2.0E-05	0.0E+00	0.0E+00	0.0E+00	0.0E+00	0.0E+00	0.0E+00	0.0E+00	0.0E+00
	Glutamate dehydrogenase (NADP+) [EC:1.4.1.4]	0.0E+00	0.0E+00	0.0E+00	4.1E-06	0.0E+00	0.0E+00	0.0E+00	0.0E+00	0.0E+00	0.0E+00	0.0E+00	0.0E+00
	Glutaminase [EC:3.5.1.2]	7.2E-05	0.0E+00	0.0E+00	0.0E+00	0.0E+00	0.0E+00	0.0E+00	0.0E+00	0.0E+00	0.0E+00	0.0E+00	0.0E+00
	Glutamate synthase (NADPH/NADH) [EC:1.4.1.13 1.4.1.14]	0.0E+00	1.5E-04	6.1E-04	4.1E-06	0.0E+00	1.0E-04	0.0E+00	0.0E+00	0.0E+00	0.0E+00	0.0E+00	0.0E+00
	Asparagine synthase (glutamine-hydrolysing) [EC:6.3.5.4]	0.0E+00	0.0E+00	0.0E+00	2.7E-04	0.0E+00	0.0E+00	0.0E+00	0.0E+00	0.0E+00	0.0E+00	0.0E+00	0.0E+00
	Nitrilase [EC:3.5.5.1]	0.0E+00	0.0E+00	0.0E+00	3.7E-05	0.0E+00	5.1E-04	0.0E+00	0.0E+00	0.0E+00	0.0E+00	0.0E+00	0.0E+00
	Electron transfer flavoprotein beta subunit	0.0E+00	1.5E-04	0.0E+00	0.0E+00	0.0E+00	0.0E+00	0.0E+00	0.0E+00	0.0E+00	0.0E+00	2.6E-06	0.0E+00
**Sulfur metabolism**	3'-phosphoadenosine 5'-phosphosulfate synthase [EC:2.7.7.4 2.7.1.25]	0.0E+00	0.0E+00	0.0E+00	4.1E-06	0.0E+00	0.0E+00	0.0E+00	0.0E+00	0.0E+00	0.0E+00	0.0E+00	0.0E+00
	Adenylylsulfate kinase [EC:2.7.1.25]	0.0E+00	0.0E+00	1.4E-03	3.8E-04	0.0E+00	0.0E+00	0.0E+00	0.0E+00	0.0E+00	0.0E+00	0.0E+00	0.0E+00
	Sulfate adenylyltransferase [EC:2.7.7.4]	0.0E+00	0.0E+00	0.0E+00	2.4E-05	0.0E+00	0.0E+00	0.0E+00	0.0E+00	0.0E+00	0.0E+00	0.0E+00	0.0E+00
	Phosphoadenosine phosphosulfate reductase [EC:1.8.4.8]	0.0E+00	0.0E+00	0.0E+00	5.3E-05	0.0E+00	0.0E+00	0.0E+00	0.0E+00	0.0E+00	0.0E+00	0.0E+00	0.0E+00
	Sulfite reductase (NADPH) flavoprotein alpha-component [EC:1.8.1.2]	0.0E+00	0.0E+00	0.0E+00	0.0E+00	0.0E+00	0.0E+00	0.0E+00	0.0E+00	2.1E-04	0.0E+00	0.0E+00	0.0E+00
	Cystathionine beta-lyase [EC:4.4.1.8]	0.0E+00	0.0E+00	0.0E+00	6.5E-05	0.0E+00	0.0E+00	0.0E+00	0.0E+00	0.0E+00	0.0E+00	0.0E+00	0.0E+00
	Cystathionine gamma-synthase [EC:2.5.1.48]	0.0E+00	0.0E+00	0.0E+00	1.2E-05	0.0E+00	0.0E+00	0.0E+00	0.0E+00	0.0E+00	0.0E+00	0.0E+00	0.0E+00
	Homoserine O-succinyltransferase [EC:2.3.1.46]	0.0E+00	0.0E+00	0.0E+00	1.6E-05	0.0E+00	0.0E+00	0.0E+00	0.0E+00	0.0E+00	0.0E+00	0.0E+00	0.0E+00
	Homoserine O-acetyltransferase [EC:2.3.1.31]	1.4E-04	2.3E-03	0.0E+00	2.0E-05	0.0E+00	0.0E+00	0.0E+00	0.0E+00	0.0E+00	0.0E+00	0.0E+00	0.0E+00
	Cysteine synthase A [EC:2.5.1.47]	0.0E+00	0.0E+00	4.6E-04	0.0E+00	0.0E+00	1.0E-04	0.0E+00	0.0E+00	0.0E+00	0.0E+00	0.0E+00	0.0E+00
