# Supplementary material for: A viability-linked metagenomic analysis of cleanroom environments: eukarya, prokaryotes, and viruses
Source: Microbiome. 2015 Dec 8;3:62. doi: 10.1186/s40168-015-0129-y (PMC4672508; doi:10.1186/s40168-015-0129-y)
Supplement: Additional file 5: Figure S3. — A PDF figure showing significantly different KEGG Orthologs (KOs). 35 KEGG Orthologs that showed significant differences between the six comparison groups. (PDF 39 kb) [file 40168_2015_129_MOESM5_ESM.pdf]

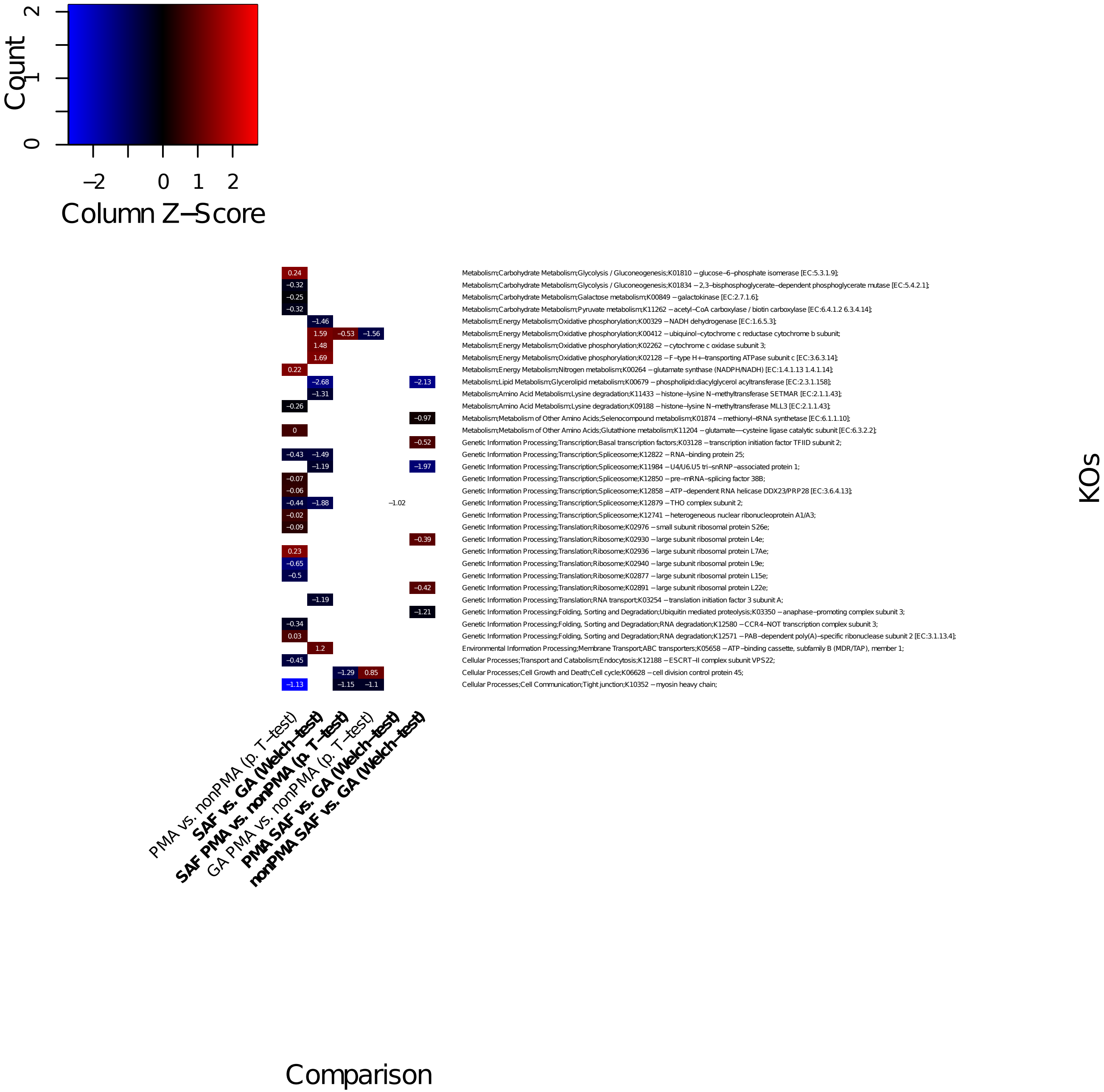

Count
0
1
2
−2
0
1
2
Column Z−Score
0.24
−0.32
−0.25
−0.32
−1.46
1.59
−0.53
−1.56
1.48
1.69
0.22
−2.68
−2.13
−1.31
−0.26
−0.97
0
−0.52
−0.43
−1.49
−1.19
−1.97
−0.07
−0.06
−0.44
−1.88
−1.02
−0.02
−0.09
−0.39
0.23
−0.65
−0.5
−0.42
−1.19
−1.21
−0.34
0.03
1.2
−0.45
−1.29
0.85
−1.13
−1.15
−1.1
Metabolism;Carbohydrate Metabolism;Glycolysis / Gluconeogenesis;K01810 −glucose−6−phosphate isomerase [EC:5.3.1.9];
Metabolism;Carbohydrate Metabolism;Glycolysis / Gluconeogenesis;K01834 −2,3−bisphosphoglycerate−dependent phosphoglycerate mutase [EC:5.4.2.1];
Metabolism;Carbohydrate Metabolism;Galactose metabolism;K00849 −galactokinase [EC:2.7.1.6];
Metabolism;Carbohydrate Metabolism;Pyruvate metabolism;K11262 −acetyl−CoA carboxylase / biotin carboxylase [EC:6.4.1.2 6.3.4.14];
Metabolism;Energy Metabolism;Oxidative phosphorylation;K00329 −NADH dehydrogenase [EC:1.6.5.3];
Metabolism;Energy Metabolism;Oxidative phosphorylation;K00412 −ubiquinol−cytochrome c reductase cytochrome b subunit;
Metabolism;Energy Metabolism;Oxidative phosphorylation;K02262 −cytochrome c oxidase subunit 3;
Metabolism;Energy Metabolism;Oxidative phosphorylation;K02128 −F−type H+−transporting ATPase subunit c [EC:3.6.3.14];
Metabolism;Energy Metabolism;Nitrogen metabolism;K00264 −glutamate synthase (NADPH/NADH) [EC:1.4.1.13 1.4.1.14];
Metabolism;Lipid Metabolism;Glycerolipid metabolism;K00679 −phospholipid:diacylglycerol acyltransferase [EC:2.3.1.158];
Metabolism;Amino Acid Metabolism;Lysine degradation;K11433 −histone−lysine N−methyltransferase SETMAR [EC:2.1.1.43];
Metabolism;Amino Acid Metabolism;Lysine degradation;K09188 −histone−lysine N−methyltransferase MLL3 [EC:2.1.1.43];
Metabolism;Metabolism of Other Amino Acids;Selenocompound metabolism;K01874 −methionyl−tRNA synthetase [EC:6.1.1.10];
Metabolism;Metabolism of Other Amino Acids;Glutathione metabolism;K11204 −glutamate−−cysteine ligase catalytic subunit [EC:6.3.2.2];
Genetic Information Processing;Transcription;Basal transcription factors;K03128 −transcription initiation factor TFIID subunit 2;
Genetic Information Processing;Transcription;Spliceosome;K12822 −RNA−binding protein 25;
Genetic Information Processing;Transcription;Spliceosome;K11984 −U4/U6.U5 tri−snRNP−associated protein 1;
Genetic Information Processing;Transcription;Spliceosome;K12850 −pre−mRNA−splicing factor 38B;
Genetic Information Processing;Transcription;Spliceosome;K12858 −ATP−dependent RNA helicase DDX23/PRP28 [EC:3.6.4.13];
Genetic Information Processing;Transcription;Spliceosome;K12879 −THO complex subunit 2;
Genetic Information Processing;Transcription;Spliceosome;K12741 −heterogeneous nuclear ribonucleoprotein A1/A3;
Genetic Information Processing;Translation;Ribosome;K02976 −small subunit ribosomal protein S26e;
Genetic Information Processing;Translation;Ribosome;K02930 −large subunit ribosomal protein L4e;
Genetic Information Processing;Translation;Ribosome;K02936 −large subunit ribosomal protein L7Ae;
Genetic Information Processing;Translation;Ribosome;K02940 −large subunit ribosomal protein L9e;
Genetic Information Processing;Translation;Ribosome;K02877 −large subunit ribosomal protein L15e;
Genetic Information Processing;Translation;Ribosome;K02891 −large subunit ribosomal protein L22e;
Genetic Information Processing;Translation;RNA transport;K03254 −translation initiation factor 3 subunit A;
Genetic Information Processing;Folding, Sorting and Degradation;Ubiquitin mediated proteolysis;K03350 −anaphase−promoting complex subunit 3;
Genetic Information Processing;Folding, Sorting and Degradation;RNA degradation;K12580 −CCR4−NOT transcription complex subunit 3;
Genetic Information Processing;Folding, Sorting and Degradation;RNA degradation;K12571 −PAB−dependent poly(A)−specific ribonuclease subunit 2 [EC:3.1.13.4];
Environmental Information Processing;Membrane Transport;ABC transporters;K05658 −ATP−binding cassette, subfamily B (MDR/TAP), member 1;
Cellular Processes;Transport and Catabolism;Endocytosis;K12188 −ESCRT−II complex subunit VPS22;
Cellular Processes;Cell Growth and Death;Cell cycle;K06628 −cell division control protein 45;
Cellular Processes;Cell Communication;Tight junction;K10352 −myosin heavy chain;
KOs
PMA vs. nonPMA (p. T−test)
SAF vs. GA (Welch−test)
SAF PMA vs. nonPMA (p. T−test)
GA PMA vs. nonPMA (p. T−test)
PMA SAF vs. GA (Welch−test)
nonPMA SAF vs. GA (Welch−test)
Comparison
