# Supplementary material for: A viability-linked metagenomic analysis of cleanroom environments: eukarya, prokaryotes, and viruses
Source: Microbiome. 2015 Dec 8;3:62. doi: 10.1186/s40168-015-0129-y (PMC4672508; doi:10.1186/s40168-015-0129-y)
Supplement: Additional file 7: S1. — A PDF file containing the R script MCStats1.2. R script for statistical analysis of ecological community data. (DOC 25 kb) [file 40168_2015_129_MOESM7_ESM.pdf]

```
# source.R
library(GUniFrac)
library(vegan)
library(ape)
library(gplots)
library(mgcv)

OUTPUT <- file(paste(comm_file,"_analysis_overview.txt",sep=""),
open="wt")
sink(OUTPUT)
sink(OUTPUT, type="message")

dat=read.table(comm_file, sep="\t", row.names=1, header=T)
meta=read.table(meta_file1, sep="\t", row.names=1, header=T)
meta2=read.table(meta_file2, sep="\t", row.names=1, header=T)
dist_matrix = vegdist(t(dat), method="bray")

#
======================================================================
===================================
# Dump log file
#
======================================================================
===================================

if (Rarefy) {
cat("\nFiltering your data with Rarefication to the lowest number of
OTU abundance in the samples. Iterations were set to: \n", Iterations,
"\n")
cat("Output-file is:",
paste(comm_file,"_",Iterations,"_","_bray.txt",sep=""), "\n")
} else {
cat("\nRarefication is not activated\n")}
if (runANOVA) {
cat("\nFiltering your data with ANOVA. Filter category is: \n",
catANOVA, "\np-value is: ", ANOVApVal, "\n")
cat("Output-file is:", paste(comm_file,"_ANOVA_tbl.txt",sep=""), "\n")
} else {
cat("\nANOVA filtering was not activated.\n")}
if (readDIST) {
    cat("\nUsing pre-computed distance matrix\n")
} else {
    cat("\nNo distance matrix was read.\n")}
if (runPCoA) {
cat("\nPerforming PCoA analysis:\n")
cat("Output-file is:", paste(comm_file,"_PCoA.pdf",sep=""), "\n")
} else {
cat("\nPCoA analysis was omitted\n")}
if (runNMDS) {
cat("\nPerforming NMDS:\n")
```

```
cat("Output-file is:", paste(comm_file,"_NMDS.pdf",sep=""), "\n")
} else {
cat("\nNMDS analysis was omitted\n")}
if (runHC) {
cat("\nPerforming HC analysis\n")
cat("Output-file is:", paste(comm_file,"_HC.pdf",sep=""), "\n")
} else {
cat("\nHC was omitted\n")}
if (runMRPP) {
cat("\nPerforming MRPP based on: ", groupingMRPP, "\n")
cat("Output-file is:", paste(comm_file,"_MRPP.txt",sep=""), "\n")
} else {
cat("\nMRPP was omitted\n")}
if (runAdonis_cat) {
cat("\nPerforming Adonis based on: ", catAdonis, "(categorical)\n")
cat("Output-file is:", paste(comm_file,"_Adonis_cat.txt",sep=""),
"\n")
} else {
cat("\nAdonis on catergorical variable was omitted\n")}
if (runAdonis_cont) {
cat("\nPerforming Adonis based on: ", contAdonis, "(numerical)\n")
cat("Output-file is:", paste(comm_file,"_Adonis_cont.txt",sep=""),
"\n")
} else {
cat("\nAdonis on continous variable was omitted\n")}
if (runBioENV) {
cat("\nRunning BioENV on complete meta2 file (continous variables)\n")
cat("Output-file is:", paste(comm_file,"_BioENV.txt",sep=""), "\n")
} else {
cat("\nBioENV was omitted\n")
}
if (runNMDSvec) {
cat("\nPerforming NMDS with vector overlay (bioplotting)\n")
cat("Output-files are:", paste(comm_file,"_NMDSvec.pdf, ",sep=""),
paste(comm_file,"_NMDSvec.txt",sep=""), "\n")
} else {
cat("\nNMDS with vector overlay was omitted\n")
}
if (runNMDScurve) {
cat("\nPerforming NMDS with vector overlay (bioplotting)\n")
cat("Output-file is:", paste(comm_file,"_NMDScurve.pdf",sep=""))
} else {
cat("\nNMDS with curve-fitting was omitted\n")
}
if (runHeatmap) {
cat("\nPerforming Heatmap\n")
cat("Output-file is:", paste(comm_file,"_Heatmap.pdf",sep=""), "\n")
} else {
cat("\nHeatmap was omitted\n")
}
```

```
if (HM_dend_spec) {
cat("\nPerforming Heatmap with species dendrogram\n")
cat("Output-file is:",
paste(comm_file,"_Heatmap_dend_spec.pdf",sep=""), "\n")
} else {
cat("\nHeatmap with species dendrogram was omitted\n")
}
if (HM_dend_samp) {
cat("\nPerforming Heatmap with sample dendrogram\n")
cat("Output-file is:",
paste(comm_file,"_Heatmap_dend_samp.pdf",sep=""), "\n")
} else {
cat("\nHeatmap with sample dendrogram was omitted\n")
}
if (HM_dend_both) {
cat("\nPerforming Heatmap with sample and species dendrogram\n")
cat("Output-file is:",
paste(comm_file,"_Heatmap_dend_both.pdf",sep=""), "\n")
} else {
cat("\nHeatmap with sample and species dendrogram was omitted\n")
}
sink(type="message")
sink()

#
======================================================================
===================================
# Input and filtering
#
======================================================================
===================================
# =====================================================
# 1. Rarefication of microbiome data
if (Rarefy) {
    dat_rar = (Rarefy(t(dat)))$otu.tab.rff
    dist_collect = vegdist(dat_rar, method="bray")
    dat2 = dat_rar
    rm (dat_rar)
    for (j in 1:(Iterations-1)) {
        dat_rar = (Rarefy(t(dat)))$otu.tab.rff
        dist_collect = dist_collect + vegdist(dat_rar, method="bray")
        rm(dat_rar)
    }
    dist_final = dist_collect / Iterations
    write.table(as.matrix(dist_final), paste(comm_file,"_dist_",
Iterations, "_bray.txt", sep=""),quote=F, sep="\t", col.names = TRUE)
    dist_matrix=dist_final
    rm(dist_collect, dist_final)
    dat = dat2
}
```

```
# ====================================================
# 2. ANOVA filtering of OTUs
if (runANOVA) {
	meta_col_names <- colnames(meta)
	i=1
	for (i in 1:length(meta[1,]))
	{
		if (catANOVA[1] == meta_col_names[i]) {
			type <- meta[,i]
			is.factor(type)
		}}
	aovFunc=function(x)
	{
	y=data.frame(type,x);
	anova(aov(x ~ type, y))
	}
	aovResults <- apply(dat, 1, aovFunc)
	pVals <- data.frame(lapply(aovResults, function(y)
{ y["Pr(>F)"][1,] }) )
	write.table(t(pVals),
paste(comm_file,"_anova_pVals.txt",sep=""), quote=F, sep='\t')
	pVals_trans  <- t(pVals)
	data_pVals <- cbind(dat,pVals_trans)
	length_tbl  <- length(t(dat)[,1])+1
	dat <- data_pVals[!(data_pVals[,length_tbl]>=ANOVApVal),]
	write.table(t(dat),
paste(comm_file,"_selected_anova_pVals.txt",sep=""), quote=F,
sep='\t')
	dat  <- dat[,-length_tbl]
    dist_matrix = vegdist(t(dat), method="bray")
}

# ====================================================
# 3. Read distance matrix
if (readDIST) {
    dist_matrix <- as.dist(read.table(comm_file, sep="\t",
row.names=1, header=T))
}



#
======================================================================
===================================
# Statistical tests
#
======================================================================
===================================
```

```
# =====================================================
# 1. Richness calculation (Shannon-Wiener index)
if (runShannonWiener) {
shan_ind=diversity(t(dat), index = "shannon", MARGIN = 1, base =
exp(1))
write.table(shan_ind, paste(comm_file,"_ShannonWiener.txt",sep=""),
sep="\t")
}

# =====================================================
# 2. STANDARD ORDINATIONS
# === PCoA ===
if (runPCoA) {
PCoA=pcoa(dist_matrix, correction="none", rn=NULL)
PCoAAxis1=(PCoA$values[1,1])/sum(PCoA$values[,1])
PCoAAxis2=(PCoA$values[2,1])/sum(PCoA$values[,1])
PCoAsum=PCoAAxis1+PCoAAxis2
        PCoA_out <- file(paste(comm_file,"_PCoA.txt",sep=""),
open="wt")
        sink(PCoA_out)
        sink(PCoA_out, type="message")
        print("Explained variance axis 1:")
        print(PCoAAxis1)
        print("Explained variance axis 2:")
        print(PCoAAxis2)
        print("Explained for both axis:")
        print(PCoAsum)
        sink(type="message")
        sink()
pdf(paste(comm_file,"_PCoA.pdf",sep=""))
biplot(PCoA, Y=NULL, plot.axes = c(1,2), dir.axis1=1, dir.axis2=1,
rn=NULL)
dev.off()}
# === NMDS ===
if (runNMDS) {
        mds=metaMDS(dist_matrix,wascores=FALSE,zerodist="add")
        mds
        mds$points
        mds.points <- cbind(mds$points[,1:2])
        mds.points
        mds.dat <- cbind(mds.points, meta)
        mds.dat
        pdf(paste(comm_file,"_NMDS.pdf",sep=""))
        plot(mds.points, xlab="NMDS1", ylab="NMDS2",
main=paste("Stress = ",mds$stress,sep=""))
        attach(mds.dat)
        meta_col_names <- colnames(meta)
        i=1
        for (i in 1:length(meta[1,]))
        {
```

```
                if (groupingNMDS[1] == meta_col_names[i]) {
                        text(mds.dat[1:2], labels=mds.dat[,i+2],
col="black", pos=4)
                }
        }
        dev.off()
        detach(mds.dat)}

# =====================================================
# 3. HIERARCHIAL CLUSTERING
# === HC - AN ===
if (runHC) {
        clust_dist<-hclust(dist_matrix, "average")
        pdf(paste(comm_file,"_HC.pdf",sep=""))
        plot(clust_dist)
        dev.off()
}

# =====================================================
# 4. SIGNIFICANCE TESTING OF ENVIRONMENTAL FACTORS
# === MRPP ===
if (runMRPP) {
        meta_col_names <- colnames(meta)
        i=1
        for (i in 1:length(meta[1,]))
        {
                if (groupingMRPP[1] == meta_col_names[i]) {
                MRPP_val<-mrpp(dist_matrix, meta[,i],
permutations=999)
                mrpp_out <-
file(paste(comm_file,"_",groupingMRPP,"_MRPP.txt",sep=""), open="wt")
                sink(mrpp_out)
                sink(mrpp_out, type="message")
                cat("\ngroupingMRRP:", groupingMRPP, "\n")
                print(MRPP_val)
                sink(type="message")
                sink()
                }
        }
}
# === ADONIS (categorical) ===
if (runAdonis_cat) {
        meta_col_names <- colnames(meta)
        i=1
        for (i in 1:length(meta[1,]))
        {
                if (catAdonis == meta_col_names[i]) {
                Adonis_cat<-adonis(formula = dist_matrix ~ meta[,i],
data=meta, permutations=999)
                        Adonis_cat_out <-
```

```
file(paste(comm_file,"_",catAdonis,"_Adonis_cat.txt",sep=""),
open="wt")
                        sink(Adonis_cat_out)
                        sink(Adonis_cat_out, type="message")
                        cat("Categorical variable used: ",catAdonis,
"\n")
                        print(Adonis_cat)
                        sink(type="message")
                        sink()
                }
        }
}
# === ADONIS (continous) ===
if (runAdonis_cont) {
        meta2_col_names <- colnames(meta2)
        i=1
        for (i in 1:length(meta2[1,]))
        {
                if (contAdonis[1] == meta2_col_names[i]) {
                Adonis_cont<-adonis(formula = dist_matrix ~
meta2[,i], data=meta2, permutations=999)
                        Adonis_cont_out <-
file(paste(comm_file,"_",contAdonis,"_Adonis_cont.txt",sep=""),
open="wt")
                        sink(Adonis_cont_out)
                        sink(Adonis_cont_out, type="message")
                        cat("Continous variable used: ",contAdonis,
"\n")
                        print(Adonis_cont)
                        sink(type="message")
                        sink()
                }
        }
}
# === BIOENV ===
if (runBioENV) {
        correl_cont=bioenv(dist_matrix, meta2, method="spearman",
index="bray")
        bioenv_out <- file(paste(comm_file,"_BioENV.txt",sep=""),
open="wt")
        sink(bioenv_out)
        sink(bioenv_out, type="message")
        print(correl_cont)
        print(summary(correl_cont))
        sink(type="message")
        sink()}

# ====================================================
# 5. NMDS: Vector and curve fitting
if (runNMDSvec) {
```

```
        mds=metaMDS(dist_matrix,wascores=FALSE,zerodist="add")
        mds
        mds$points
        mds.points <- cbind(mds$points[,1:2])
        mds.points
        mds.dat <- cbind(mds.points, meta)
        mds.dat
        vecs <- envfit(mds, meta2, permu = 999)
        NMDSvec_out <- file(paste(comm_file,"_NMDSvec.txt",sep=""),
open="wt")
        sink(NMDSvec_out)
        sink(NMDSvec_out, type="message")
        print(vecs)
        sink(type="message")
        sink()
        pdf(paste(comm_file,"_NMDSvec.pdf",sep=""))
        plot(mds.points, xlab="NMDS1", ylab="NMDS2",
main=paste("Stress = ",mds$stress,sep=""))
        attach(mds.dat)
        meta_col_names <- colnames(meta)
        i=1
        for (i in 1:length(meta[1,]))
        {
                if (groupingNMDSvec[1] == meta_col_names[i]) {
                        text(mds.dat[1:2], labels=mds.dat[,i+2],
col="black", pos=4)
                }
        }
        plot(vecs, p.max = vec_pVal)
        dev.off()
        detach(mds.dat)}

if (runNMDScurve) {
        mds=metaMDS(dist_matrix,wascores=FALSE,zerodist="add")
        mds
        mds$points
        mds.points <- cbind(mds$points[,1:2])
        mds.points
        mds.dat <- cbind(mds.points, meta)
        mds.dat
        pdf(paste(comm_file,"_NMDScurve.pdf",sep=""))
        plot(mds.points, xlab="NMDS1", ylab="NMDS2",
main=paste("Stress = ",mds$stress,sep=""))
        attach(mds.dat)
        meta_col_names <- colnames(meta)
        i=1
        for (i in 1:length(meta[1,]))
        {
                if (groupingNMDScurve[1] == meta_col_names[i]) {
                        text(mds.dat[1:2], labels=mds.dat[,i+2],
```

```
col="black", pos=4)
                 }
        }
        meta2_col_names <- colnames(meta2)
        for (i in 1:length(meta2[1,]))
        {
                if (NMDScurveModel[1] == meta2_col_names[i]) {
                        surf  <- meta2[,i]
                        with(meta2, ordisurf(mds, surf, add = TRUE,
col = ColCurve))
                }
        }
        dev.off()
        detach(mds.dat)}

# ====================================================
# 6. HEATMAPS
if (runHeatmap) {
pdf(paste(comm_file,"_Heatmap.pdf",sep=""))
heatmap.2(as.matrix(dat),col=(colorpanel(4000,low=col_min,
mid=col_mid,
high=col_max)),dendrogram="none",xlab="samples",ylab="OTUs",Rowv=NA,Co
lv=NA,trace="none", scale="row") #colsep=c(5,14), sepcolor="white",
sepwidth=c(0.05,0.05)
dev.off()
}
if (HM_dend_spec) {
pdf(paste(comm_file,"_Heatmap_dend_spec.pdf",sep=""))
heatmap.2(as.matrix(dat),col=(colorpanel(4000,low=col_min,
mid=col_mid,
high=col_max)),distfun=dist,dendrogram="row",xlab="samples",ylab="OTUs
",Colv=NA,trace="none", scale="row") # scale="none"
dev.off()
}
if (HM_dend_samp) {
pdf(paste(comm_file,"_Heatmap_dend_samp.pdf",sep=""))
heatmap.2(as.matrix(dat),col=(colorpanel(4000,low=col_min,
mid=col_mid,
high=col_max)),distfun=dist,dendrogram="column",xlab="samples",ylab="O
TUs",Rowv=NA,trace="none", scale="row")
dev.off()
}
if (HM_dend_both) {
pdf(paste(comm_file,"_Heatmap_dend_both.pdf",sep=""))
heatmap.2(as.matrix(dat),col=(colorpanel(4000,low=col_min,
mid=col_mid,
high=col_max)),distfun=dist,dendrogram="both",xlab="samples",ylab="OTU
s",trace="none", scale="row")
dev.off()
}
```

q()

```
## MC_Stats Version 1.2
## For questions, please contact the author:
alexander.j.probst@gmail.com
## general convention: the sample order in the community files
(abundance, distance matrix etc) and in the metadata files are exctly
the same. The script does not determine sample IDs across files (due
to multiple reasons).
## Please cite: Weinmaier and Probst et al., 2015 Microbiome


# ANALYSIS SPECIFICATIONS
#
======================================================================
===================================

# Specify input files:
#
======================================================================
===================================

#set working directory
#setwd("D://Folder1//Folder2//") # from a windows system
setwd("/Folder1/Folder2") #from a mac OS X

#specify the files you would like to input (must be in the folder =
working directory)
# 1. Species file (columns are samples, rows are species unless you
read a distance matrix which needs to be specified below)
comm_file<-c("abundance_rank_norm.txt")
# 2. specify metadata file 1: categorical variables
meta_file1<-c("cat.txt")
# 3. specify metadata file 2: continuous variables
meta_file2<-c("cont.txt")


# Filtering of the data (hint: only activate one!):
#
======================================================================
===================================
# 1. Rarefy your microbiome data (will not affect heatmaps)
Rarefy <- TRUE
Iterations <- 10

# =====================
# 2. Significance testing per OTU
runANOVA <- FALSE                                 # ANOVA to reduce
the amount of OTUs
ANOVApVal  <- c(0.01)                     # p-value threshold
catANOVA  <- c("Type1")         # specify categorical variable (meta1)
```

```
# =====================
# 3. Read a distance matrix from previous experiment (make sure that
it is tab-delimited and the headers match the columns)
# IMPORTANT: Since this input will not provide OTU abundances, please
do not use heatmap functions or shannon wiener index below.
readDIST <- TRUE

# Statistical tests
#
=======================================================================
===================================
# 1. Diversity richness measure: Shannon-Wiener index (cannot be
calculated from distance matrix only)
runShannonWiener <- FALSE

# =====================
# 2. Standard ordinations (NMDS, PCAs and PCoAs)
runPCoA <- TRUE
# =
runNMDS <- TRUE                                   # based on bray-
curtis dissimilarity
groupingNMDS <- c("name")        # specify categorical variable
(meta1)

# =====================
# 3. Hierarchial clustering analysis
runHC <- TRUE

# =====================
# 4. Significance testing of environmental factors (MRPP, Adonis,
BioENV)
runMRPP <- FALSE
groupingMRPP <- c("name")                # specify categorical
variable (meta1)
# =
runAdonis_cat <- FALSE
catAdonis <- c("type1")                           # specify
categorical variable (meta1)
# =
runAdonis_cont <- FALSE
contAdonis <- c("PCR_hybe")                       # specify
categorical variable (meta2)
# =
runBioENV <- FALSE                                        # BioENV
utilizes entire meta2 file

# =====================
# 5. NMDS with metadata
runNMDSvec  <- FALSE                              # uses entire meta2
file
```

```
groupingNMDSvec <- c("type1")             # specify continuous
variable (meta2)
vec_pVal        <- c(0.05)                                # p-Value
threshold for plotting meta2 variables
# =
runNMDScurve <- FALSE
groupingNMDScurve <- c("type2")           # specify categorical
variable (meta1)
NMDScurveModel  <-       c("coverage")    # specify categorical
variable (meta2)
ColCurve  <- c("green")                           # specify color for
curve

# =====================
# 6. Heatmap
col_min  <- rgb(0, 0, 255, maxColorValue=255)
col_mid  <- c("white")
col_max  <- rgb(255, 0, 0, maxColorValue=255)
# =
runHeatmap        <- FALSE                                # no
dendrograms
# =
HM_dend_spec <- FALSE                             # based on
euclidean dissimilarity
# =
HM_dend_samp <- FALSE                             # based on
euclidean dissimilarity
# =
HM_dend_both <- FALSE                             # based on
euclidean dissimilarity

# =====================
# Source the analysis code: (specify path to code.R here)
source("/path_to_code/code.R")
```
