# Supplementary material for: A viability-linked metagenomic analysis of cleanroom environments: eukarya, prokaryotes, and viruses
Source: Microbiome. 2015 Dec 8;3:62. doi: 10.1186/s40168-015-0129-y (PMC4672508; doi:10.1186/s40168-015-0129-y)

All samples (n=12)

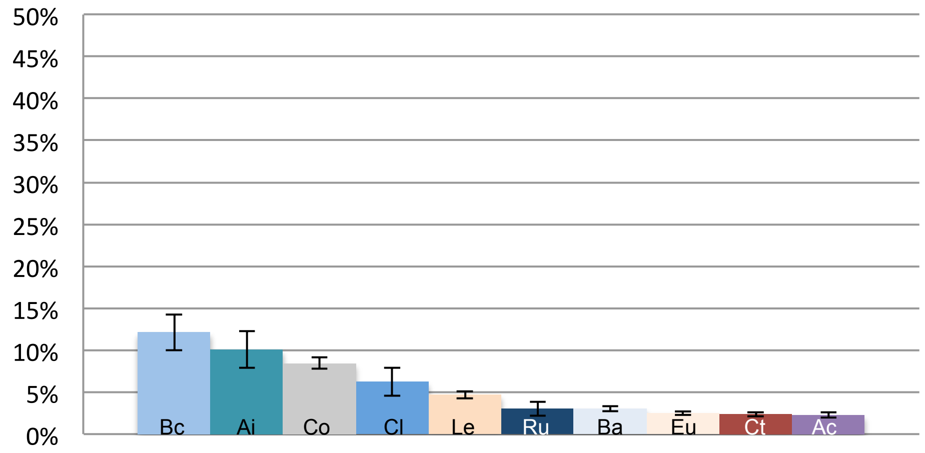

cellular organisms	(Co)	Bacteria	(Ba)
Eukaryota	(Eu)	Proteobacteria (Bac)	(Pr)
Acanthamoeba (Euk, Amoebozoa)	(Ac)	Gammaproteobacteria (Bac)	(Ga)
Leotiomyceta (Euk, Fungi)	(Le)	Enterobacteriaceae (Bac)	(En)
Exophiala (Euk, Fungi)	(Ex)	Diplorickettsia (Bac)	(Di)
Mycosphaerella (Euk, Fungi)	(My)	Rickettsiella (Bac)	(Ri)
Claviceps (Euk, Fungi)	(Ca)	Acinetobacter (Bac)	(Ai)
Boreoeutheria (Euk, Fungi)	(Bo)	Pseudomonas (Bac)	(Ps)
Simiiformes (Euk, Primates)	(Si)	Alphaproteobacteria (Bac)	(Al)
Catarrhini (Euk, Primates)	(Ct)	Bacillus (Bac)	(Bc)
Homo (Euk, Primates)	(Ho)	Clostridiales (Bac)	(Cl)
unclassified viruses	(Uv)	Ruminiclostridium (Bac)	(Ru)
unclassified Circoviridae (Vir)	(Uc)	Staphylococcus (Bac)	(St)
		Gardnerella (Bac)	(Gr)
		Propionibacterium (Bac)	(Pp)

PMA: SAF + GA (n=6)

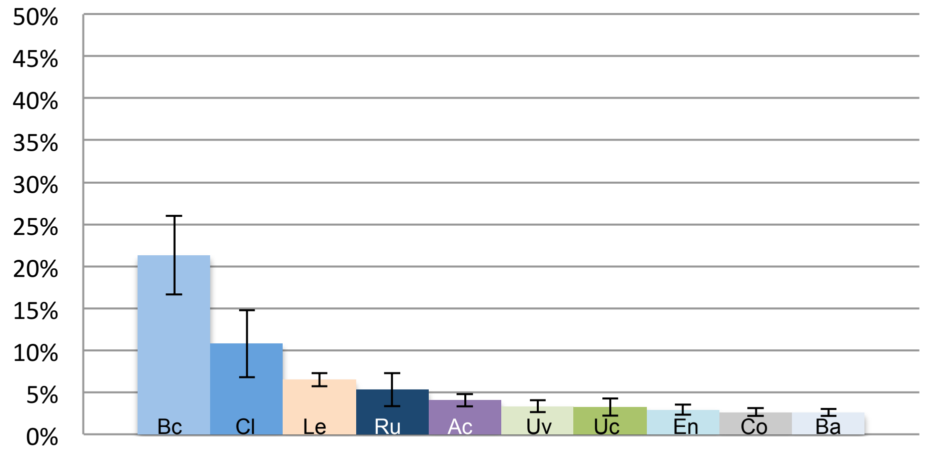

nonPMA: SAF + GA (n=6)

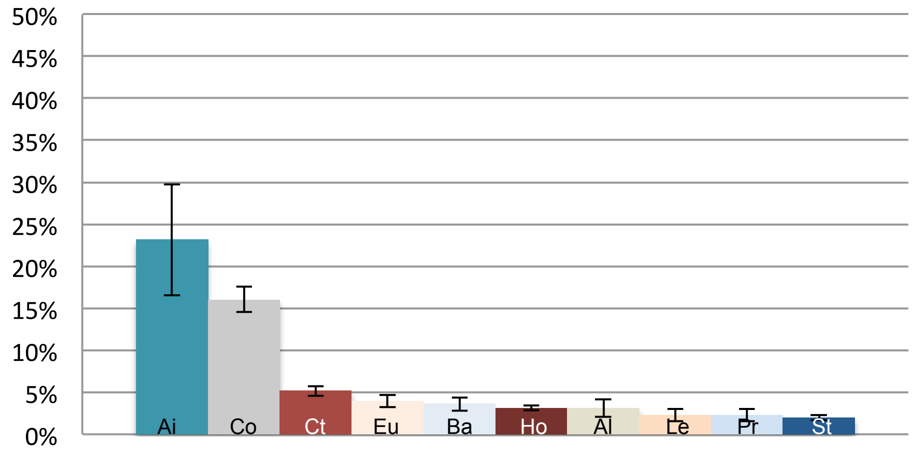

SAF: PMA + nonPMA (n=6)

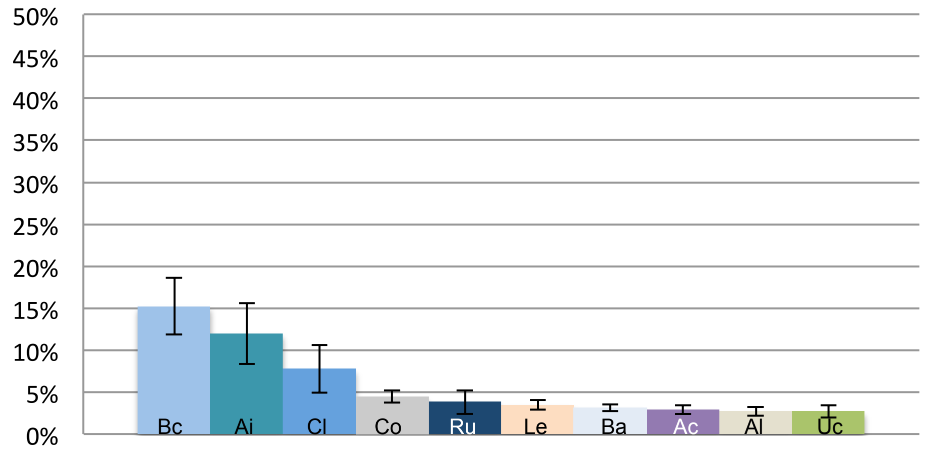

GA: PMA + nonPMA (n=6)

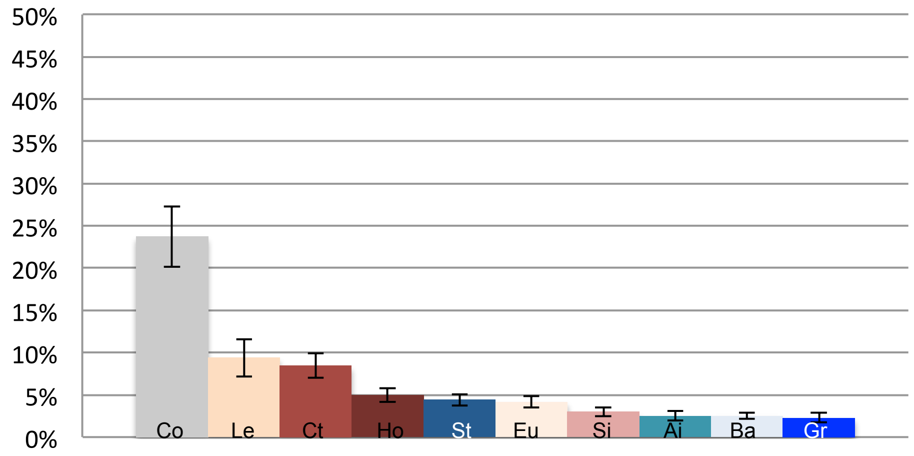

SAF_nonPMA (n=3)

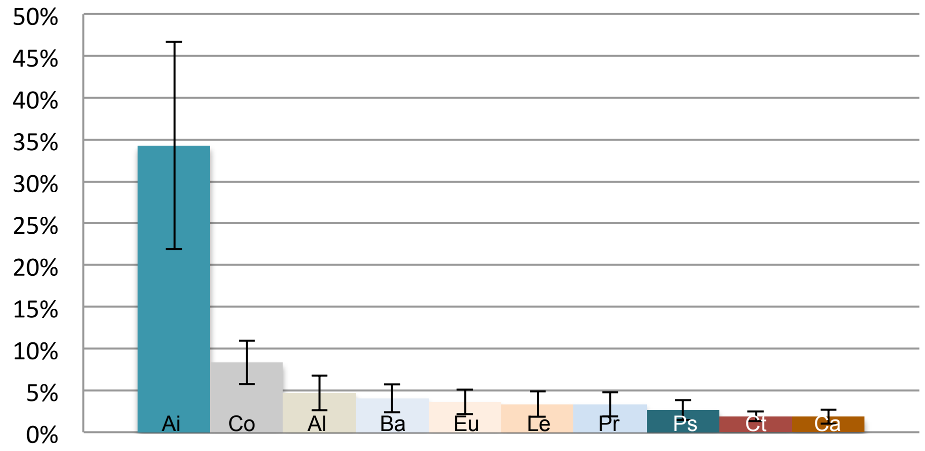

GA_nonPMA (n=3)

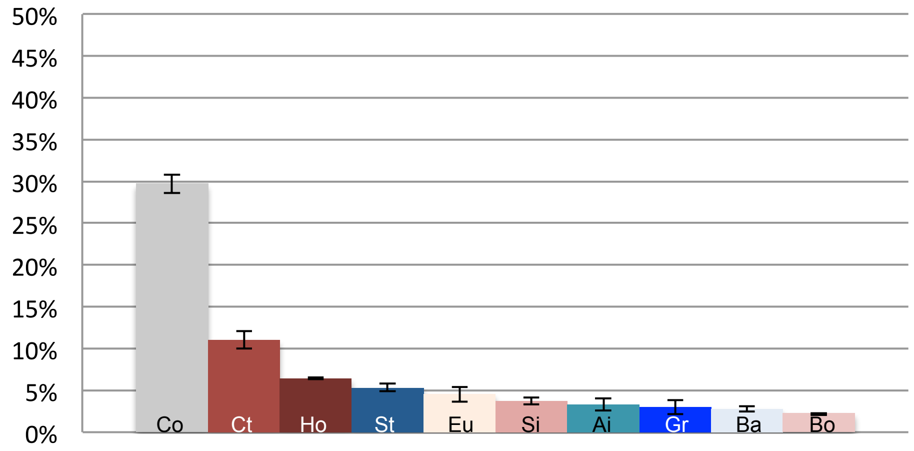

Supplement: Additional file 8: Figure S4. — A PDF figure depicting additional ranked abundance curves. Rank-abundance curves of relative abundance data in different sample groups. Absolute abundance of each taxon was normalized by the total abundance of all samples considered. Top ten taxa are listed. Error-bars indicate standard deviation. Proportional abundances of top 20 bacterial taxa (genus level) compared by sample. (PDF 162 kb) [file 40168_2015_129_MOESM8_ESM.pdf]
